# Identification of a gene cluster for telomestatin biosynthesis and heterologous expression using a specific promoter in a clean host

**DOI:** 10.1038/s41598-017-03308-5

**Published:** 2017-06-13

**Authors:** Keita Amagai, Haruo Ikeda, Junko Hashimoto, Ikuko Kozone, Miho Izumikawa, Fumitaka Kudo, Tadashi Eguchi, Takemichi Nakamura, Hiroyuki Osada, Shunji Takahashi, Kazuo Shin-ya

**Affiliations:** 1 Technology Research Association for Next Generation Natural Products Chemistry, 2-4-7 Aomi, Koto-ku, Tokyo 135-0064 Japan; 2 RIKEN Center for Sustainable Resource Science, Natural Product Biosynthesis Research Unit, 2-1 Hirosawa, Wako, Saitama 351-0198 Japan; 3 Kitasato Institute for Life Sciences, Kitasato University, 1-15-1 Kitasato, Minami-ku, Sagamihara, Kanagawa 252-0373 Japan; 4 Japan Biological Informatics Consortium, 2-4-7 Aomi, Koto-ku, Tokyo 135-0064 Japan; 5 Department of Chemistry, Tokyo Institute of Technology, 2-12-1 O-okayama, Meguro-ku, Tokyo 152-8551 Japan; 6 RIKEN Center for Sustainable Resource Science, Molecular Structure Characterization Unit, 2-1 Hirosawa, Wako, Saitama 351-0198 Japan; 7 RIKEN Center for Sustainable Resource Science, Chemical Biology Research Group, 2-1 Hirosawa, Wako, Saitama 351-0198 Japan; 8 National Institute of Advanced Industrial Science and Technology, 2-4-7 Aomi, Koto-ku, Tokyo 135-0064 Japan

## Abstract

Telomestatin, a strong telomerase inhibitor with G-quadruplex stabilizing activity, is a potential therapeutic agent for treating cancers. Difficulties in isolating telomestatin from microbial cultures and in chemical synthesis are bottlenecks impeding the wider use. Therefore, improvement in telomestatin production and structural diversification are required for further utilization and application. Here, we discovered the gene cluster responsible for telomestatin biosynthesis, and achieved production of telomestatin by heterologous expression of this cluster in the engineered *Streptomyces avermitilis* SUKA strain. Utilization of an optimal promoter was essential for successful production. Gene disruption studies revealed that the *tlsB*, *tlsC*, and *tlsO–T* genes play key roles in telomestatin biosynthesis. Moreover, exchanging TlsC core peptide sequences resulted in the production of novel telomestatin derivatives. This study sheds light on the expansion of chemical diversity of natural peptide products for drug development.

## Introduction

**Figure 1:**
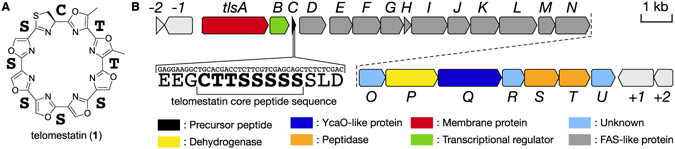
(**A**) The chemical structure of **1** and putative amino acid constitution from the structure. (**B**) Gene organization of **1** biosynthetic gene cluster found in *S. anulatus* 3533-SV4. Putative functions of each ORF in the **1** biosynthetic gene cluster are summarized in Table S1.

Natural products produced by actinomycetes are used for a variety of drugs and bioprobes for chemical biology and biochemistry research. Telomestatin (**1**) (Fig. 1A) is a macrocyclic peptide isolated from *Streptomyces anulatus* 3533-SV4^1^. Owing to specific binding to the telomeric G-quadruplex structure of the 3’-telomere end and strong telomerase inhibitory activity^2^, it is globally used as the standard substance for *in vitro* assays^3–8^. To date, various telomerase inhibitors have been isolated from natural products^9–11^ or developed in organic synthesis studies^12–14^, and their activities have been evaluated. However, no compound showing stronger activity than **1** has been found, except for synthetic (*S*)-isomer^15^.

Despite the importance for biological research applications, the supply of **1** is limited. The purification process from a culture broth of *S. anulatus* 3533-SV4 has been extremely difficult, because it produces small amounts of **1** in the presence of a variety of secondary metabolites. Total synthesis studies of **1** and its derivatives have also been performed^15–17^. However, because of technical difficulties in the isolation process, the yield was insufficient for downstream chemical modification. Therefore, the development of an efficient system for producing **1** has been desired. We adopted a heterologous gene expression system from which any produced compound can be easily recovered. The clean host strain *Streptomyces avermitilis* SUKA^18, 19^, which disrupts major secondary metabolite biosynthetic gene clusters, represents a breakthrough for the efficient production and isolation of **1**. Here, we report the identification, by heterologous expression, of the gene cluster that drives **1** biosynthesis, and the derivatization of **1** for future drug development.

## Results and Discussion

### Exploration of the 1 Biosynthetic Gene Cluster and Promoter-assisted Heterologous Expression

Based on the structure of **1**, we first assumed an involvement of non-ribosomal peptide synthetase for biosynthesis. However, genome sequence analyses of *S. anulatus* 3533-SV4 did not support this assumption. Therefore, we speculated that **1** was derived from the ribosomally synthesized and post-translationally modified peptide (RiPP) biosynthetic machinery^20, 21^, which may catalyse the formation of a single thiazoline ring from cysteine, two methyloxazole rings from threonines, and five oxazole rings from serines, via dehydration and oxidation. To discover the genes encoding the peptide backbone of **1**, we searched the whole-genome sequence of *S. anulatus* 3533-SV4. As expected, we identified the gene (*tlsC*) encoding such a core peptide sequence (CTTSSSSS) in a small open reading frame encoding 42 amino acids (Fig. 1B). Importantly, the *tlsC* gene was also associated with putative genes encoding YcaO-type dehydratase (*tlsQ*) and flavin-dependent oxidoreductase (*tlsP*) in the same operon (Table S1). Unexpectedly, the operon harboured a set of genes that are possibly involved in fatty acid biosynthesis by type-II FAS machinery, which is considered unnecessary for **1** biosynthesis.

**Figure 2:**
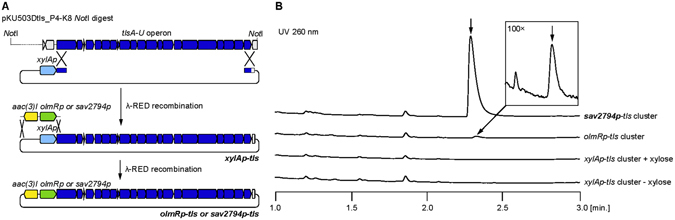
Promoter-assisted heterologous expression of **1** biosynthetic gene cluster in SUKA strain. (**A**) Overview of heterologous expression vector. (**B**) HPLC/MS analysis of metabolites produced by the SUKA17 strain, which was transformed with the *tls* gene cluster containing the *xylA*, *olmR*, and *sav2794* gene promoters. The peak from the metabolites from SUKA17 (pKU492Acos::*sav2794p::tls*_cluster) showed a *m/z* ratio of 583 [M+H]^+^, corresponding to the authentic **1** molecule.

To identify the gene cluster responsible for the biosynthesis of **1**, we performed heterologous gene expression using the engineered host strain *Streptomyces avermitilis* SUKA^18, 19^. First, the primary clone (pKU503Dtls_P4-K8) carrying the entire presumptive gene cluster for **1** biosynthesis was obtained from a bacterial artificial chromosome (BAC) library of S. *anulatus* 3533-SV4. The operon containing 21 genes was subcloned, following *Not*I restriction endonuclease digestion of pKU503Dtls_P4-K8. Then, a xylose-inducible promoter (*xylAp*), originating from the *xylA* (*sav7182*) promoter of *S. avermitilis*, was inserted into the construct, upstream of the **1** gene cluster (Fig. 2A). The resulting construct was integrated into a specific locus of the *S. avermitilis* SUKA17^18^ chromosome via polyethylene glycol-assisted protoplast transformation. The transformants were cultured in the presence of 2% xylose, and their metabolite profiles were analysed by HPLC/MS. However, the production of **1** was not observed (Fig. 2B), indicating that the *xylA* promoter was not suitable for activation of the **1** gene cluster. Next, we considered that transcription should be controlled during the productive phase of the secondary metabolites. Therefore, we replaced *xylAp* with *olmRp* and *sav2794p*, respectively (Fig. 2A). The *olmRp* promoter drives expression of the *olmRI* (*sav2902*) gene, which encodes the LuxR-family transcriptional regulator controlling the expression of genes required for oligomycin biosynthesis in *S. avermitilis* ^22^, and the transcription was found to begin during the late logarithmic phase of growth. The *sav2794p* promoter drives expression of the *sav2794* gene, which encodes a secreted neutral metalloprotease and was expected to be expressed during the late logarithmic phase of growth. These two promoters were introduced upstream of the **1** gene cluster via λ-RED recombination^23^. The resulting constructs were integrated into *S. avermitilis* SUKA17, and their metabolite profiles were analysed using HPLC/MS (Fig. 2B). We detected only a trace amount of **1** under the control of the *olmRp*. When the *sav2794p* was used for expression of the **1** gene cluster, we detected a clear and discrete peak of **1**, with a pseudo molecular ion peak having an *m/z* ratio of 583 [M + H]^+^. The yield of **1** reached more than 5 mg/L in 0.3 × BPS medium^24^. This was the first demonstration that this specific promoter (*sav2794p*) is essential for production of this secondary metabolite using heterologous gene expression.

### Gene Inactivation Experiments Determined Essential Genes for 1 Biosynthesis

**Figure 3:**
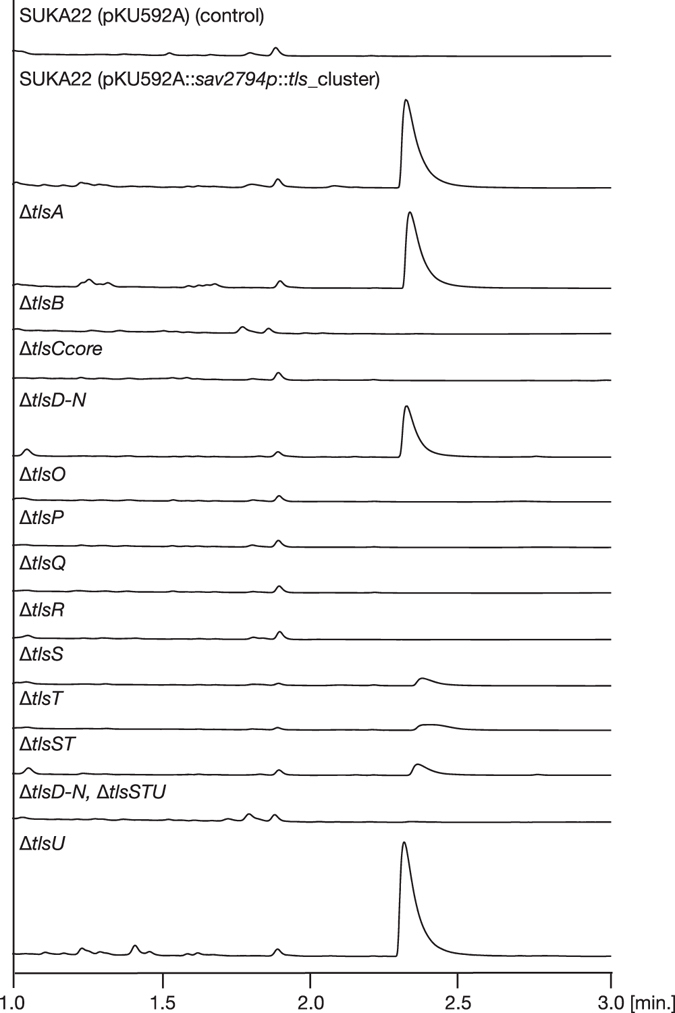
HPLC/MS analysis of metabolites produced by the SUKA22 strain, which was transformed with *tls* gene cluster containing each indicated tls gene deletion.

Our success with producing **1** at a high yield enabled us to characterize the functions of the genes involved in **1** biosynthesis. The gene cluster contains genes that potentially function as a transporter (*tlsA*), a transcriptional regulator (*tlsB*), and a precursor peptide (*tlsC*). This gene cluster also encodes unidentified enzymes that are homologous to enzymes promoting fatty acid biosynthesis (from *tlsD* to *tlsN*), post-translational peptide modification (*tlsP* and *tlsQ*), and peptidase function (*tlsS*, and *tlsT*); furthermore, it also encodes molecules homologous to proteins with unknown functions (*tlsO*, *tlsR*, and *tlsU*) (Fig. 1B, Table S1). To address the roles of these genes in **1** biosynthesis, we first constructed a *tlsC* disruptant (Δ*tlsCcore*), which lacks the core peptide sequence. When the Δ*tlsCcore* construct was integrated into SUKA17, it abolished the production of **1**, suggesting that the core peptide is modified by the RiPP biosynthetic machinery (Fig. 3).

Next, *tls* gene-deletion mutants were constructed using the pKU592A::*sav2794p*::*tls* cluster (Fig. S1, Tables S2 and S3). Each deletion construct was introduced into *S. avermitilis* SUKA22^19^ (isogenic to SUKA17), and the resulting metabolite profiles were analysed by HPLC/MS (Fig. 3). To examine the role of the putative transporter, we also evaluated transformants lacking the *tlsA* gene (Δ*tlsA*). Contrary to our expectation, wild type *tls* and Δ*tlsA* transformants accumulated **1** in cells, and we observed no alterations in production of **1**. However, we could not rule out involvement of the *tlsA* gene in the biosynthesis of **1**, because the growth of the Δ*tlsA* transformants was slower than that of the wild type. Transformants lacking the *tlsB* gene (Δ*tlsB*) showed complete abolishment of **1** production, suggesting that TlsB is involved in **1** biosynthesis as a positive transcriptional regulator. The C-terminal sequence of TlsB contains a helix-turn-helix motif, which is widely observed in the LuxR family of transcriptional regulators. Moreover, TlsB showed homology to AviC1, which regulates avilamycin A biosynthesis^25^, and Lipreg1, which regulates lipomycin biosynthesis^26^. Consistent with our finding, deletion of the *aviC1* and *lipreg1* genes resulted in a significant loss in production of the corresponding metabolites. Compared to cells with wild type *tls*, the transformants lacking the genes *tlsD* through *tlsN* (Δ*tlsD–N*) showed diminished production of **1** (Fig. 3 and Fig. S2). Metabolite analysis revealed that the production of a compound with a pseudo molecular ion peak at *m/z* 298 [M + H]^+^, termed FA297, was abolished in Δ*tlsD–N* transformants (Fig. S3). In addition, the production of **1** and FA297 was concomitantly abolished in Δ*tlsB* mutants (Fig. S3). The compositional formula of FA297 was determined to be C_20_H_43_N by HRESIMS (Fig. S4). As FA297 was turned out to be a mixture, further characterization of chemical entity was attempted using GC/MS (Fig. S5). Based on the molecular formula and the GC/MS data, aliphatic amine structures were suggested. However, the chemical structure could not be determined because of low abundance. Next, we constructed Δ*tlsS*, Δ*tlsT*, and Δ*tlsST* transformants to examine whether they accumulated biosynthetic precursors, because TlsS and TlsT are annotated as peptidases that might be involved in cyclic peptide formation by cleavage of the leader peptide moiety of precursor peptides. Contrary to our expectation, each mutant showed production of **1**, although their production levels were significantly reduced (Fig. 3, Fig. S2); this indicated that peptide precursors of **1** were partially modified by intrinsic peptidases, even in the absence of TlsS and TlsT. To address the roles of TlsD-N during peptide modification, we also constructed a transformant lacking the *tlsD*–*N* and *tlsSTU* genes. Interestingly, the mutant showed completely abolished production of **1**. Based on these observations, we speculated that a presence of FA297 may be involved in correct processing of biosynthetic intermediates. In addition, based on translational coupling between the *tlsT* and *tlsU* genes, we expected the *tlsU* gene to be involved in **1** production. However, single deletion of the *tlsU* gene did not affect the production of **1**.

**Figure 4:**
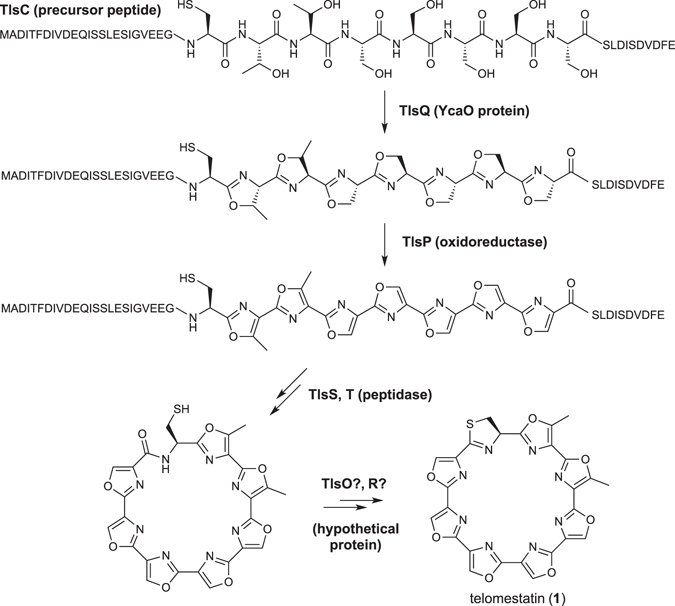
Proposed biosynthetic pathway for **1**.

To analyse the biosynthetic mechanism of peptide modification, transformants lacking the *tlsO, tlsP, tlsQ*, or *tlsR* genes (Δ*tlsO*, Δ*tlsP*, Δ*tlsQ*, or Δ*tlsR*) were constructed, and the resulting metabolites were analysed by HPLC/MS (Fig. 3). TlsQ shared homology with the YcaO protein, which is generally involved in formation of the azoline ring in RiPP biosynthesis. TlsP is homologous to FMN-dependent oxidoreductase, which generally catalyses azole ring formation^20^. Therefore, we predicted that the Δ*tlsP* and Δ*tlsQ* transformants would accumulate azoline and precursor peptide intermediates, respectively (Fig. 4). However, both transformants showed completely abolished production of **1** and its biosynthetic intermediates (Fig. 3). Next, we investigated TlsO and TlsR, which showed no homologies to functionally annotated proteins (Table S1). Because the Δ*tlsO* and Δ*tlsR* transformants displayed completely abolished production of **1**, and no biosynthetic intermediates accumulated, we were unable to make informed speculations regarding their respective functions (Fig. 3). *In vitro* analysis using purified enzymes is key for elucidating the functions of novel enzymes such as TlsO and TlsR. Based on a report that McbB-D is involved in microcin B17 biosynthesis^27^, a possible explanation is that TlsO, TlsP, TlsQ, and TlsR might form a heterooligomer, and that all four enzymes are indispensable for the required catalytic activities.

To gain insight into the catalytic functions of TlsO and TlsR, we compared the homologous protein sequences involved in the biosynthesis of YM-216391, which also has a head-to-tail heterocyclic ring (Fig. S6), as found in **1** ^28^. Blast analysis showed that the C-terminal sequence of TlsO shares moderate homology with the N-terminal sequence of YmBC, which is annotated to be a cyclodehydratase domain. In addition, TlsR is homologous to YmB1, which is also reported to be a cyclodehydratase. These data suggested that future characterizations of TlsO and TlsR will disclose a common modification mechanism for natural macrocyclic peptide products.

Finally, the series of *tls* gene-deletion experiments suggested that eight genes (*tlsB, C, O, P, Q, R, S*, and *T*) are essential for **1** biosynthesis (Fig. 4). The core structure of **1** was derived from the *tlsC* gene encoding the small peptide sequence. We speculate that the core peptide sequence is cyclized by TlsP and TlsQ, resulting in oxazoline/oxazole formation. TlsO and TlsR are presumably involved in formation of the last thiazoline moiety, which might occur after macrocyclisation. TlsS and TlsT may excise the N-terminal leader peptide and the C-terminal follower peptide, to form **1**.

### Creation of Novel 1 Derivatives by Amino Acid Substitution of TlsC Core Peptide Sequence

**Figure 5:**
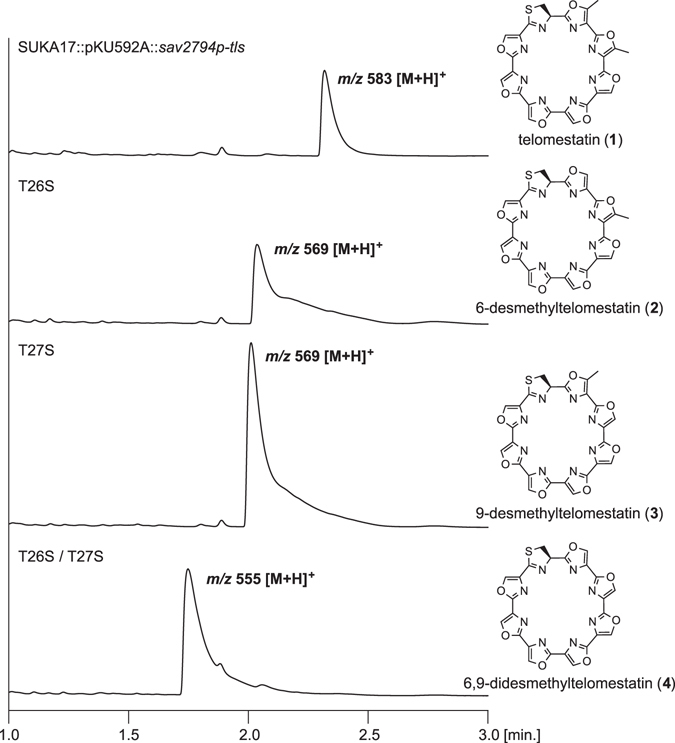
HPLC/MS analysis of metabolites produced in SUKA17, which was transformed with the *tls* gene cluster containing a mutation in *tlsC*.

It is extremely important to expand the chemical diversity of **1**, and/or to optimize production of the natural peptide itself, for future drug development. Since a binding to a telomeric G-quadluprex structure requires an isometric macrocyclic product, we established a system to produce **1** derivatives by introducing mutations into the *tlsC* gene. We introduced mutations in the same gene locus to ensure optimum expression of the *tls* gene cluster. Then, we constructed SUKA17 transformants that harboured T26S, T27S, and T26S/T27S mutations in the *tlsC* gene, and successfully produced metabolites showing *m/z* 569 [M + H]^+^ and *m/z* 555 [M + H]^+^ (Fig. 5). The analysis of collision-induced dissociation (CID) MS/MS spectra (Figs S7–13, Tables S4–5) supported the biogenetically deduced structures of **2**, **3**, and **4**. Briefly, characteristic sulphur-containing product ions were observed at *m/z* 168 from **1** and *m/z* 154 from T26S variant, whereas virtually no *m/z* 168 or *m/z* 154 peaks were observed in the CID spectra of T27S variant or T26S/T27S variant due to the loss of methyl group on second oxazole adjacent to the possible initial cleavage site (Fig. S13, more detailed discussions in the supplementary information).

Ruffner *et al*. constructed a random trunkamide core-peptide library^29^ and generated a vast number of trunkamide derivatives; however, the amount of each product was very limited, which impeded evaluations of their biological activities. Tianero *et al*. recently succeeded in producing patellins, with a yield of ~27 mg/L, from *E. coli* by feeding the bacteria cysteine and mevalonate^30^. Biosynthetic production of **1** via genetic engineering has been a challenging project, which we know because we were the group to first identify **1** ^1^. Here, we significantly improved the method of producing **1** and its derivatives by selecting an optimum host and promoter. Since we found a method of creating **1** derivatives efficiently, our future research will focus on evaluations of the biological activities of those derivatives.

In summary, we succeeded in the heterologous expression of the **1** gene cluster in the versatile host *S. avermitilis* SUKA, under the control of the *sav2794* promoter. When **1** was produced, cell grew slowly because of a stress. Our strategy of using this specific promoter provided insights into the 1) optimum expression of a gene cluster to achieve secondary metabolite biosynthesis, and 2) production of metabolites after normal cell growth in the heterologous expression host strain. Moreover, we demonstrated the efficient use of this system for expanding the chemical diversity of valuable, natural peptide products.

## Methods

### Chemicals and reagents

Ampicillin, kanamycin, and chloramphenicol were purchased from Nacalai (Kyoto Japan). Neomycin, gentamicin, and apramycin were purchased from Sigma-Aldrich Co. (MO, USA). All other chemicals were of analytical grade. Fortimicin sulphate was kindly supplied from Kyowa Hakko Co. (Tokyo, Japan). Restriction enzymes were purchased from Takara Bio, Inc. (Shiga, Japan) and New England BioLabs (MA, USA).

### DNA manipulation

The vectors and strains used in this study are summarized in Table S2. General DNA manipulations in *E. coli* were performed according to standard protocols^31^. The hot alkaline method was followed for low-copy plasmid isolation^32^. Polymerase chain reactions (PCRs) were performed using a C1000 or T100 thermal cycler (Bio-Rad). When performing λ-RED recombination, In Fusion cloning (Takara Bio, Inc.) and Gibson assembly (New England BioLabs), DNA fragments were obtained using either Phusion DNA polymerase (New England BioLabs), the Expand High Fidelity PLUS PCR system (Roche Diagnostics, Tokyo, Japan), or Pfu Ultra II Fusion HS DNA polymerase (Agilent Technologies). PCR reaction mixtures were prepared based on the manufacturers’ protocols, with minor modifications (see supplementary methods).

### Construction of a BAC library, screening, and minimization of the 1 biosynthetic gene cluster

Genome sequencing of *S. anulatus* 3533-SV4^1^ was performed using a PacBio RS II (Pacific Biosciences, Menlo Park, CA, USA), and the sequence data was assembled using HGAP2 (Pacific Biosciences). The BAC library of *S. anulatus* 3533-SV4 was constructed as described previously^19^. The pKU503Dtls_P4-K8 vector, which encodes the CTTSSSSS peptide sequence, was isolated by PCR screening. The putative **1** biosynthetic gene cluster was minimized for heterologous expression in the *S. avermitilis* SUKA strain. The 5′-terminal 528 bp and the 3′-terminal 549 bp regions found at the ends of the putative cluster were amplified from the pKU503Dtls_P4-K8 vector, using the tls-5frg_F/tls-5frg_R and tls-3frg_F/tls-3frg_R primer sets, respectively (Table S3). The gene fragments were cloned into a vector amplified from pKU492Acos_*aac*(*3*)*IV*, using the 492_F/492xylA_R primer set (Table S3), via In-Fusion® cloning with a *Spe*I site in the middle of the fragments. The *Spe*I-digested plasmid and *Not*I-digested pKU503Dtls_P4-K8 vector were both introduced into *E. coli* BW25113/pKD78 for λ-RED recombination, as described previously^23^. After a 1-d incubation at 37 °C on an LB plate containing 150 µg/mL apramycin, the desired clone was selected after colony PCR, and was re-introduced into *E. coli* DH5α to obtain pKU492Acos::*xylAp-tls*.

### Construction of promoter cassettes

The 837-bp DNA sequence (*olmRp:* the promoter sequence consisted of the region from 3,633,163 nt to 3,633,834 nt of the *S. avermitilis* genome) in the upstream region of the *olmRI* (*sav2902*) gene was amplified from a BAC clone (pKU503DoliP15-N14) using the olmRp-aacI_F and olmRp-460_R primer sets. The fragment (except for the *rpsJ* promoter) was amplified from pKU460::*rpsJp-aac3*(*I*)^18^ using the 460-olmRp-F/aacI-olmRp_R primer set. The DNA fragments were assembled via In-Fusion cloning to obtain pKU460::*olmRp-aac*(*3*)*I*. pKU460::*sav2794p* (promoter sequence consisted of the region from 3,427,321 to 3,427,705 nt of *S. avermitilis* genome) *-aac*(*3*)*I* and pKU460::ssmp*p (the promoter sequence of metalloendoprotease in *S. cinnamoeus* was obtained from the region from 870 to 1,285 nt of sequence accession #AB189036, with the introduction of a point mutation at C1252T to increase expression levels) *-aac*(*3*)*I* vectors were constructed from pKU460.

### Promoter exchange of *tls* cluster

To evaluate the effects of promoters on **1** production, the xylose-inducible promoter (*xylAp*) region in pKU492Acos::*xylAp-tls* was exchanged with the *olmR* and *sav2794* promoters. Cassettes containing the promoters and the *aac*(*3*)*I* gene were amplified from pKU460::*olmRp-aac*(*3*)*I* and pKU460::*sav2794p-aac*(*3*)*I* using either the Univ2_F/olmRp-tls_R primer set for *olmRp-aac*(*3*)*I*, or the Univ2_F/sav2794p-tls_R primer set for *sav2794p-aac*(*3*)*I*. Each promoter cassette was introduced into *E. coli* BW25113/pKD78 harbouring pKU492Acos::*xylAp*-*tls* via electroporation, and was cultured overnight on LB plates containing 25 µg/mL of fortimicin and 150 µg/mL of apramycin at 37 °C. The desired clones were selected after colony PCR, and were cultured in LB medium containing 25 µg/mL of fortimicin and 50 µg/mL of apramycin. The isolated cosmids were introduced into *E. coli* DH5α. DNA sequencing was performed to confirm that the correct promoter exchanges had occurred. The cosmids were designated as pKU492Acos::*olmRp*-*tls* and pKU492Acos::*sav2794p*-*tls*.

### Deletion of the *tls* genes

pKU492Acos::*sav2794p*-*tls* was digested with *Nhe*I and *Hin*dIII, and was then ligated into the low-copy vector pKU592A, which possesses a p15A origin of replication and the *aph*(*3’*)*II* of Tn*5* as a selectable marker, to obtain pKU592A::*sav2794p*-*tls*. The *aph*(*3’*)*II* gene in pKD13 was replaced with the *aac*(*3*)*IV* gene from pKU492Acos_*aac*(*3*)*IV* via λ-RED recombination. The pKD13-Apr_F and pKD13-Apr_R primer set was used to obtain pKD13_*aac*(*3*)*IV*, which contains the *FRT-aac*(*3*)*IV-FRT* cassette. The *aac*(*3*)*I* gene in the pKU460::*sav2794p-aac*(*3*)*I* and pKU460::ssmp*p-aac(*3*)*I* vectors was replaced with the *FRT-aac*(*3*)*IV-FRT* gene cassette, which was amplified using the 460aacI-aacIV_F and 460aacI-aacIV_R primer set (Table S3). The resulting vectors [pKU460::*sav2794p-FRT-aac*(*3*)*IV-FRT* and pKU460::ssmp*p-FRT-aac(*3*)*IV-FRT*] were used to obtain PCR fragments for *tls* gene deletion. The sets of deletion primers used are also listed in Table S3. During the process of *tls* gene deletion, the constitutively-expressed ssmp* promoter was inserted into downstream genes to assure transcription of the *tls* operon (Fig. S1A). The resulting plasmid was designated as pKU592A::*sav2794p*-*tls* Δ*tls* (Table S2). For the deletion of the genes from *tlsD* through *tlsN*, they were replaced with the ampicillin-resistance gene amplified by PCR from pGEM-3zf, using the delFAS-blac_F and delFAS-blac_R primer set, via λ-RED recombination. After *Spe*I digestion, a synthetic gene fragment encoding the 3′-UTR of *tlsC* and the 5′-UTR of *tlsO* (generated by annealing NoScar-delFAS_F and NoScar-delFAS_R) (Table S3) was joined by Gibson assembly (Fig. S1B).

### Transformation of *S. avermitilis* SUKA and isolation of metabolites

Desired plasmids harbouring the **1** biosynthetic gene cluster were prepared from *E. coli* GM2929 *hsdS*::Tn*10* to obtain unmethylated DNA constructs. The unmethylated plasmids were introduced into *S. avermitilis* SUKA17 or SUKA22 by polyethylene glycol-assisted protoplast transformation^19^. Transformants were screened by colony PCR. The desired clones were cultured in 10 mL of Tryptic Soy Broth (TSB) medium containing appropriate antibiotic(s), at 28 °C, for 2–3 d, with rotation (250 rpm) in test tubes (24-mm diameter). Because of slow growth, 1 mL of pre-culture was transferred to 10 mL of production medium (TSB, GY^33^, Q, or 0.3 × BPS^24^). After culture for 4 d at 28 °C with rotation (250 rpm), the resultant culture medium was directly sonicated at room temperature (TOMY UD-200, 30 s operation, 20 s interval, 4 times), and extracted twice with 10 mL of *n*-butanol (*n*-BuOH). The organic layer was concentrated by centrifugal evaporation using a GeneVac EZ-2 plus evaporator, and the resultant residues were dissolved in 1 mL of methanol (MeOH). After removing insoluble particles by centrifugation (20,630 × *g*, 10 min), the supernatant was filtered through a Millex-LG syringe-driven filter unit (0.20 µm, 4 mm, Millipore). The filtrate was analysed by high-performance liquid chromatography (HPLC)/MS using an ACQUITY UPLC BEH C_18_ column (1.7 µm, 2.1 × 50 mm) with CH_3_CN (30–99%), in 0.1% TFA (0–5 min) as the mobile phase, and with a flow rate of 0.65 mL/min (Figs S2 and S3).

### Creation of 1 Derivatives

pKU492Acos::*sav2794p*-*tls* was digested with *Nhe*I and *Hin*dIII, and was then ligated into pKU592A_*aac*(*3*)*IV* to obtain pKU592A*_aac*(*3*)*IV*::*sav2794p*-*tls* (Table S2). The β-lactamase gene (*bla*) with flanking *Spe*I sites was amplified from pGEM-3zf, using the primers blac-tlsCcore_F and blac-tlsCcore_R (Table S3), and the *tlsC* gene was replaced with the ampicillin-resistance gene, via λ-RED recombination, to form pKU592A*_aac*(*3*)*IV*::*sav2794p*-*tls* Δ*tlsC*::*bla*. The synthetic DNAs, which encoded the TlsC_T26S, TlsC_T27S, and TlsC_T26S/T27S mutations, were prepared by annealing each primer set (T26S_F + T26S_R, T27S_F + T27S_R, and T26S/T27S_F + T26S/T27S_R) (Table S3). After pKU592A*_aac*(*3*)*IV*::*sav2794p*::*tls* Δ*tlsC*::*bla* was digested with *Spe*I, the mutated *tlsC* fragments were assembled by Gibson assembly. After confirmation of the DNA sequences, the resulting plasmids were designated as pKU592A*_aac*(*3*)*IV*::*sav2794p*-*tls* T26S, pKU592A*_aac*(*3*)*IV*::*sav2794p*-*tls* T27S and pKU592A*_aac*(*3*)*IV*::*sav2794p*-*tls* T26S/T27S. pKU592A*_aac*(*3*)*IV*::*sav2794p*-*tls* Δ*tlsCcore* was also constructed during this process (Table S2). The plasmids were introduced into *S. avermitilis* SUKA 17 or SUKA22, and metabolites from SUKA transformants were analysed as described in the supplementary methods.

## Electronic supplementary material

Supplementary Information
