# Supplementary material for: Identification of a gene cluster for telomestatin biosynthesis and heterologous expression using a specific promoter in a clean host
Source: Sci Rep. 2017 Jun 13;7:3382. doi: 10.1038/s41598-017-03308-5 (PMC5469769; doi:10.1038/s41598-017-03308-5)
Supplement: Supplementary file 1 — Supplementary Information [file 41598_2017_3308_MOESM1_ESM.pdf]

Identification of a gene cluster for telomestatin biosynthesis and heterologous expression using a specific promoter in a clean host

Keita Amagai[1,2], Haruo Ikeda[3], Junko Hashimoto[4], Ikuko Kozone[4], Miho Izumikawa[4], Fumitaka Kudo[5], Tadashi Eguchi[5], Takemichi Nakamura[6], Hiroyuki Osada[7], Shunji Takahashi[2]*, and Kazuo Shin-ya[8]*

[1] Technology Research Association for Next generation natural products chemistry, 2-4-7 Aomi, Koto-ku, Tokyo 135-0064, Japan

[2] RIKEN Center for Sustainable Resource Science, Natural Product Biosynthesis Research Unit, 2-1 Hirosawa, Wako, Saitama 351-0198, Japan.

[3] Kitasato Institute for Life Sciences, Kitasato University, 1-15-1 Kitasato, Minami-ku, Sagamihara, Kanagawa 252-0373, Japan

[4] Japan Biological Informatics Consortium, 2-4-7 Aomi, Koto-ku, Tokyo 135-0064, Japan

[5] Department of Chemistry, Tokyo Institute of Technology, 2-12-1 O-okayama, Meguro-ku, Tokyo 152-8551, Japan

[6] RIKEN Center for Sustainable Resource Science, Molecular Structure Characterization Unit, 2-1 Hirosawa, Wako, Saitama 351-0198, Japan

[7] RIKEN Center for Sustainable Resource Science, Chemical Biology Research Group, 2-1 Hirosawa, Wako, Saitama 351-0198, Japan

[8] National Institute of Advanced Industrial Science and Technology, 2-4-7 Aomi, Koto-ku, Tokyo 135-0064, Japan

*E-mail addresses: shunjitaka@riken.jp and k-shinya@aist.go.jp

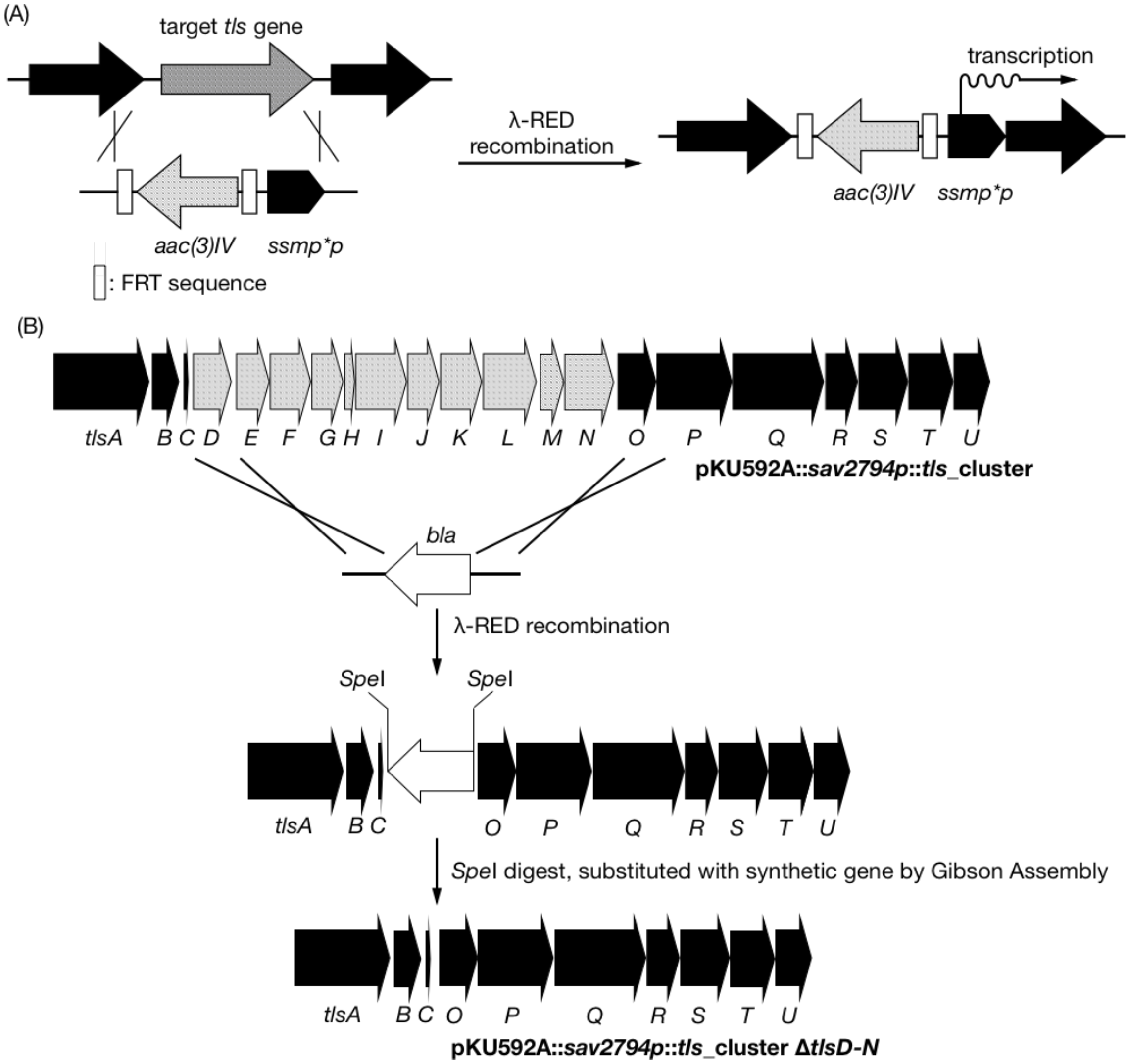

Figure S1 Construction of a heterologous-expression vector, which included the *tls* gene(s) deletion. (A) The *tls* gene was replaced with the *ssmp*p-FRT-aac(3)IV-FRT* gene cassette by λ-RED recombination. (B) Deletion of genes *tlsD* through *tlsN* was achieved by replacing them with the ampicillin-resistance gene. After *Spe*I digestion, a synthetic gene fragment encoding the 3'-UTR of *tlsC* and the 5'-UTR of *tlsO* was joined by Gibson assembly.

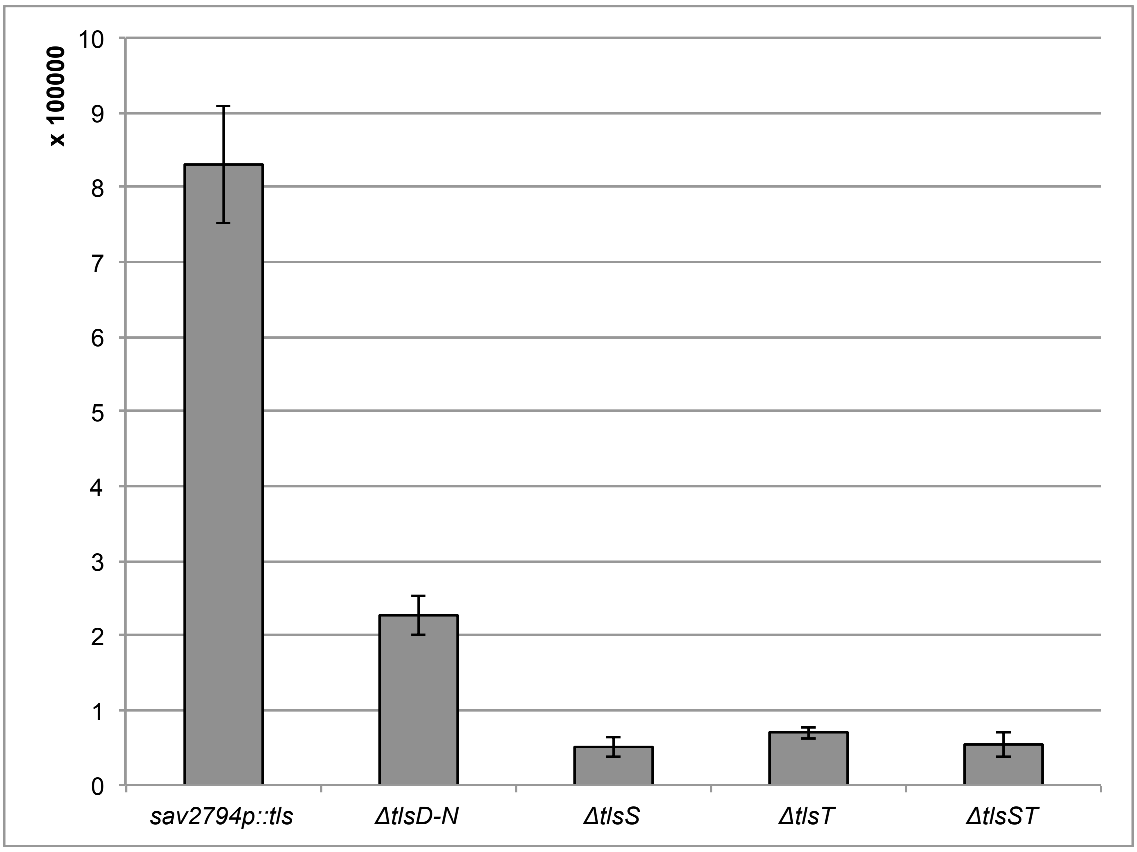

Figure S2. Comparison of the production levels of **1** from the wild type *sav2794p-tls* gene cluster and each indicated gene-knockout construct. The y-axis indicates the peak area for **1,** observed at 260 nm, after HPLC analysis.

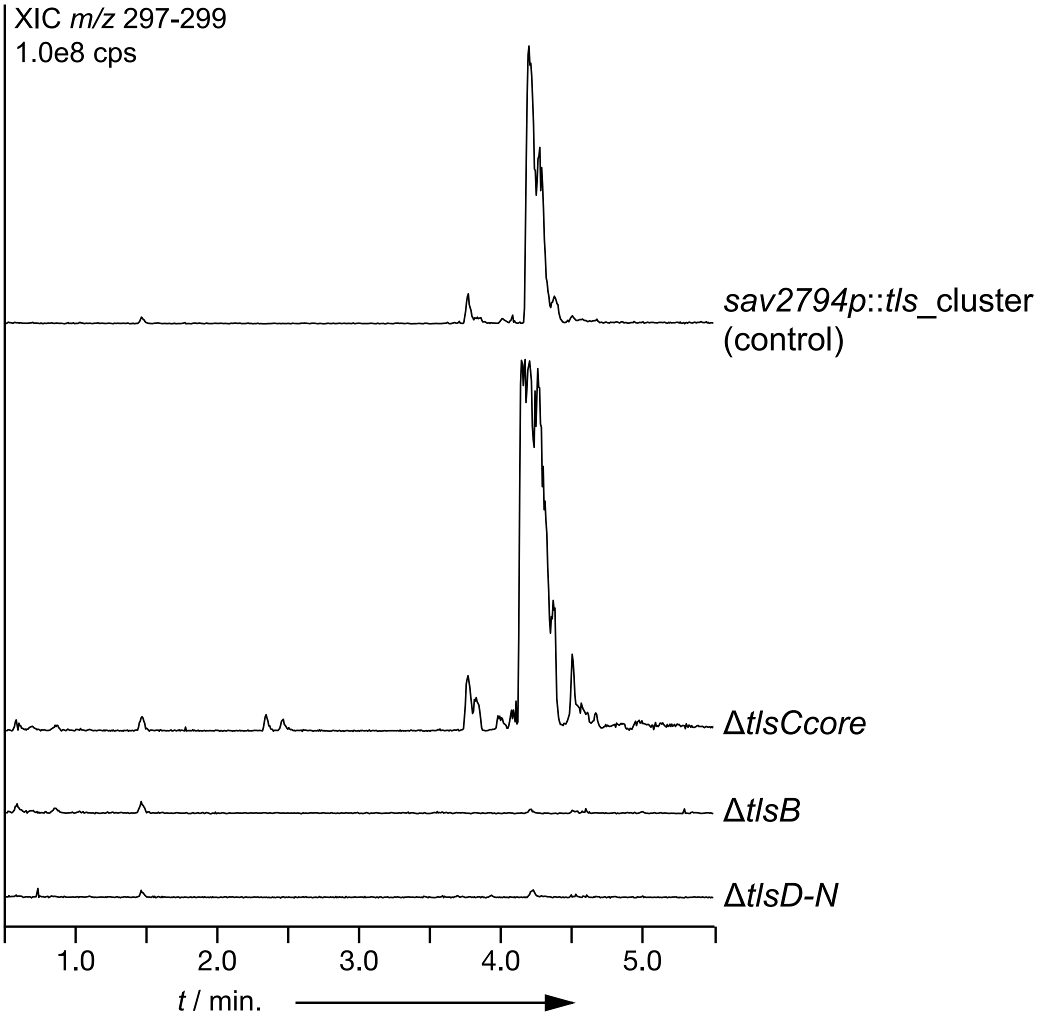

Figure S3. HPLC analysis of metabolites produced by SUKA transformants. Extracts from transformants with *sav2794p-tls*, or lacking *tlsCcore*, *tlsB*, and *tlsD* through *tlsN* genes, were analysed. Extracted ion chromatograms show peaks with *m/z* ratios ranging 297–299.

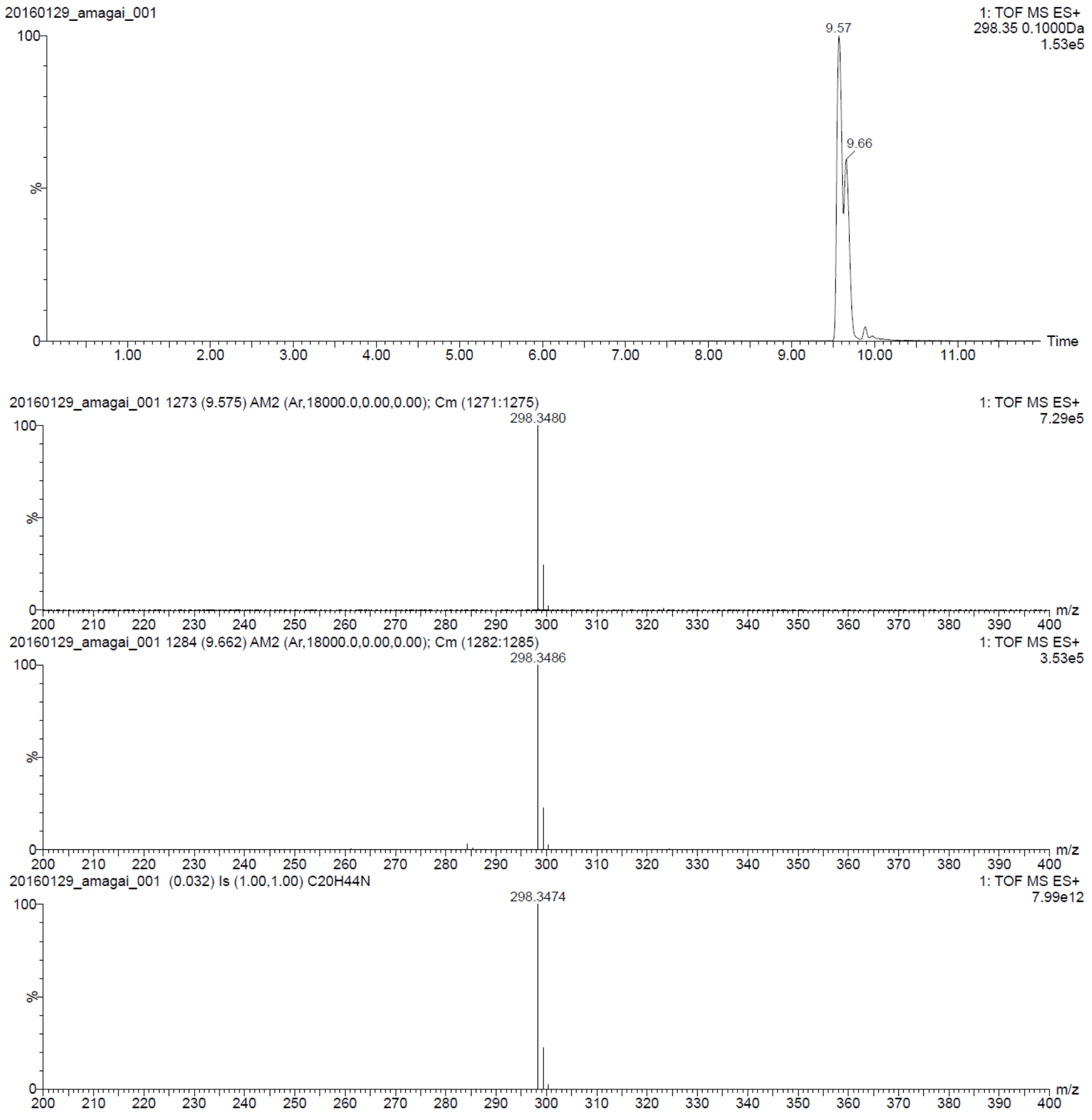

Figure S4. High-resolution HPLC/MS analysis of FA297 containing crude sample. Top panel: Extracted-ion chromatogram for *m/z* 298 (protonated molecule of FA297). 2nd panel: High-resolution mass spectrum of the main peak at 9.57 min. 3rd panel: High-resolution mass spectrum of the minor peak at 9.66 min. Bottom panel: Simulated mass spectrum for the protonated molecule of an amine with molecular formula of $C_{20}H_{43}N$.

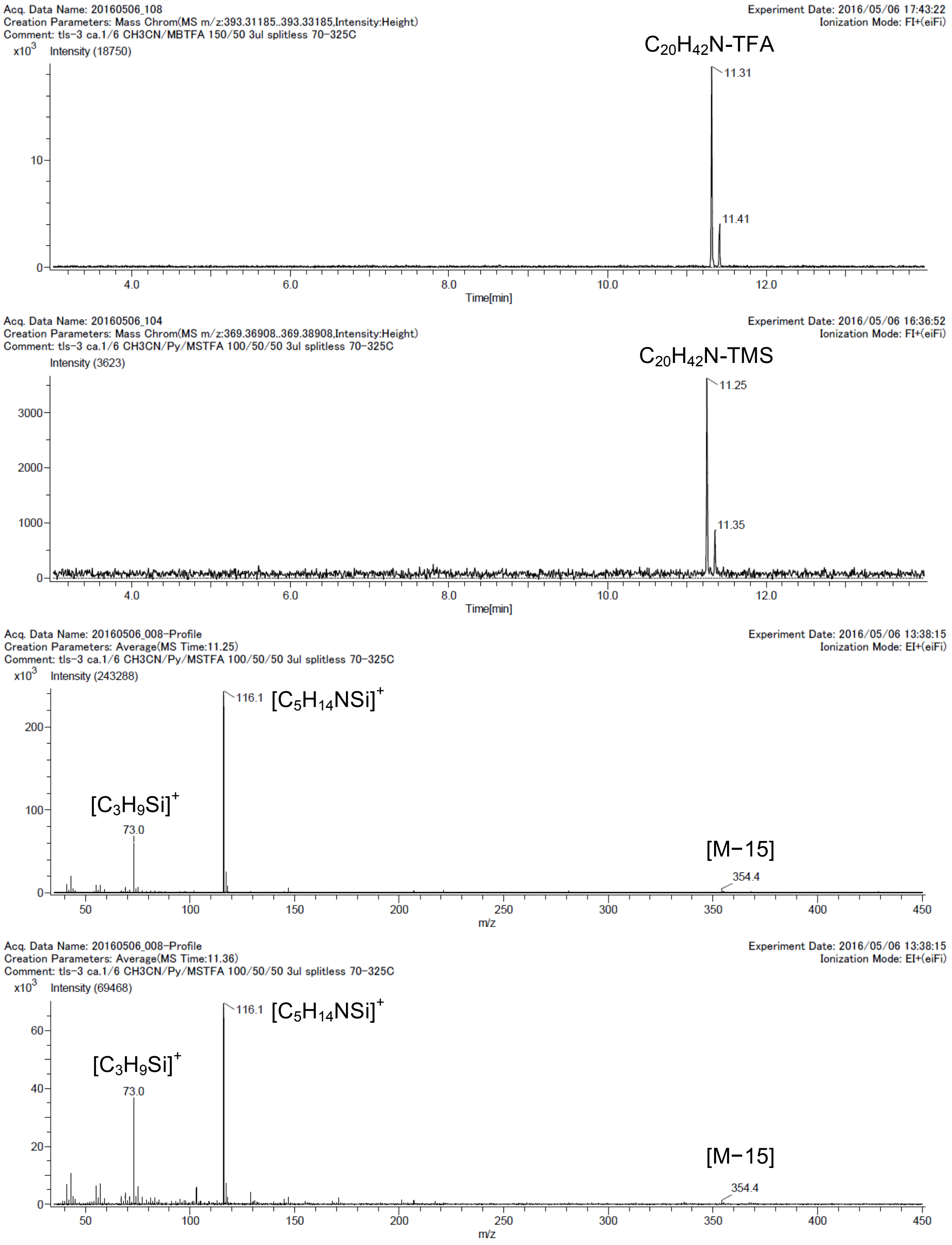

Figure S5. GC/MS analysis of FA297 containing crude sample. Top and 2nd panels: Extracted-ion chromatograms in FI mode for *m*/*z* 393 and 369 (molecular ion of TFA or TMS derivative of C-20 amines). 3rd and bottom panels: EI mass spectra of TMS-derivative at 11.25 and 11.35 min.

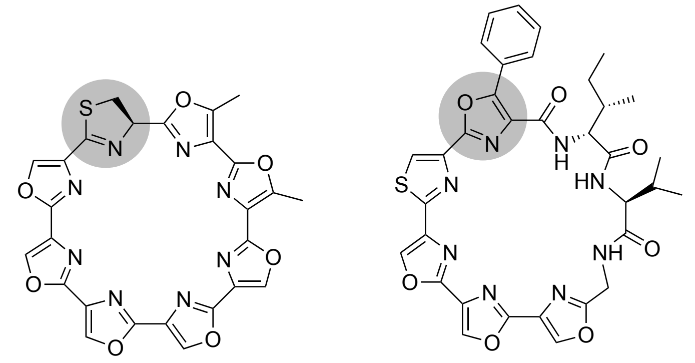

telomestatin (**1**)
CTTSSSSSS

YM-216391 (**5**)
FIVGSSSC

Figure S6. Comparison of chemical structures of **1** and YM-216391. Gray circles show head-to-tale heterocyclic rings.

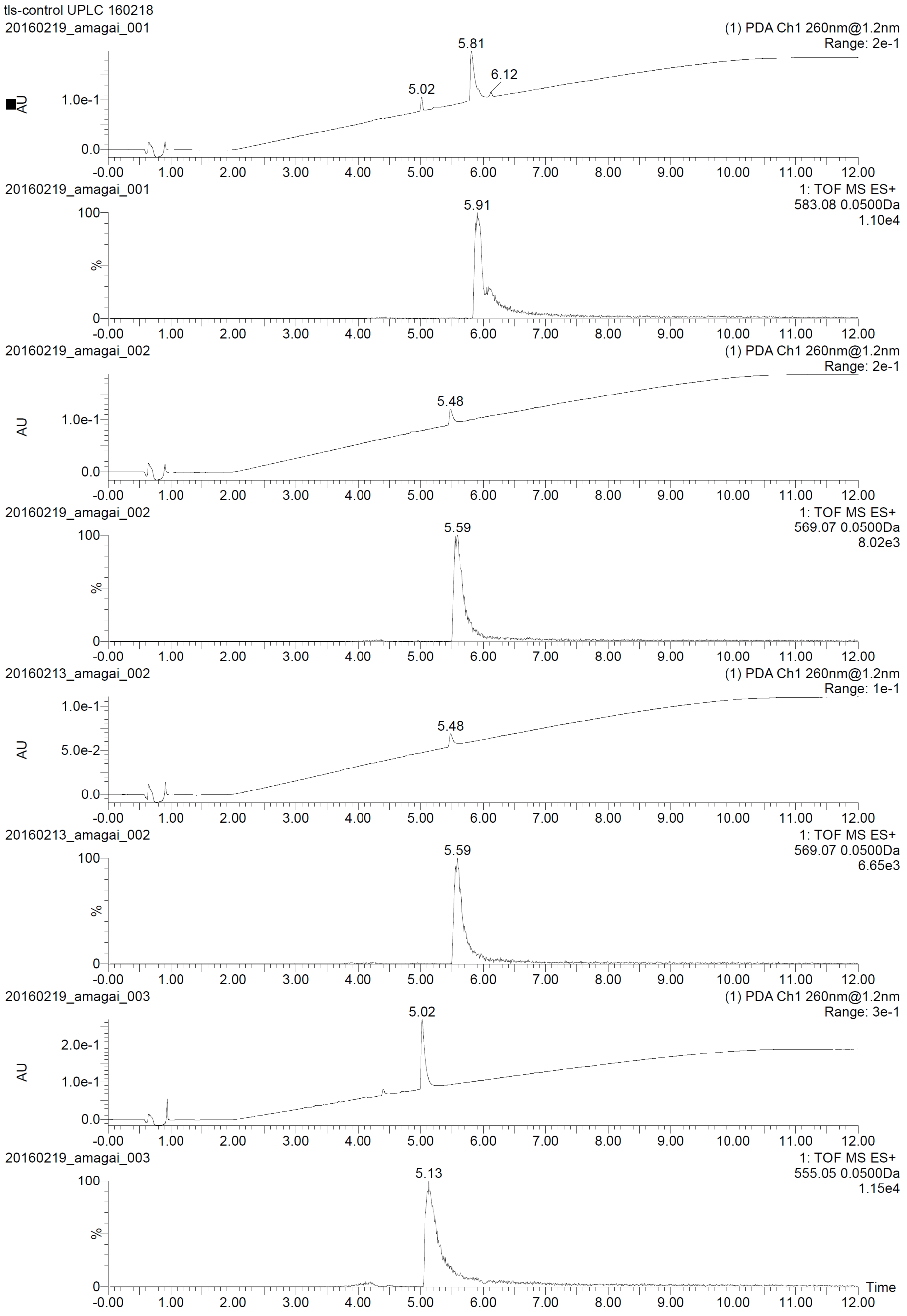

Figure S7. HPLC/MS chromatograms of purified **1**, **2**, **3**, and **4**. From top to bottom, the UV traces (260 nm) and extracted-ion chromatograms for the predicted protonated molecules for **1**, **2**, **3**, and **4** are shown. The approximate delays of 0.1 min in the extracted-ion chromatograms are attributable to the flow line between the UV cell and the electrospray probe.

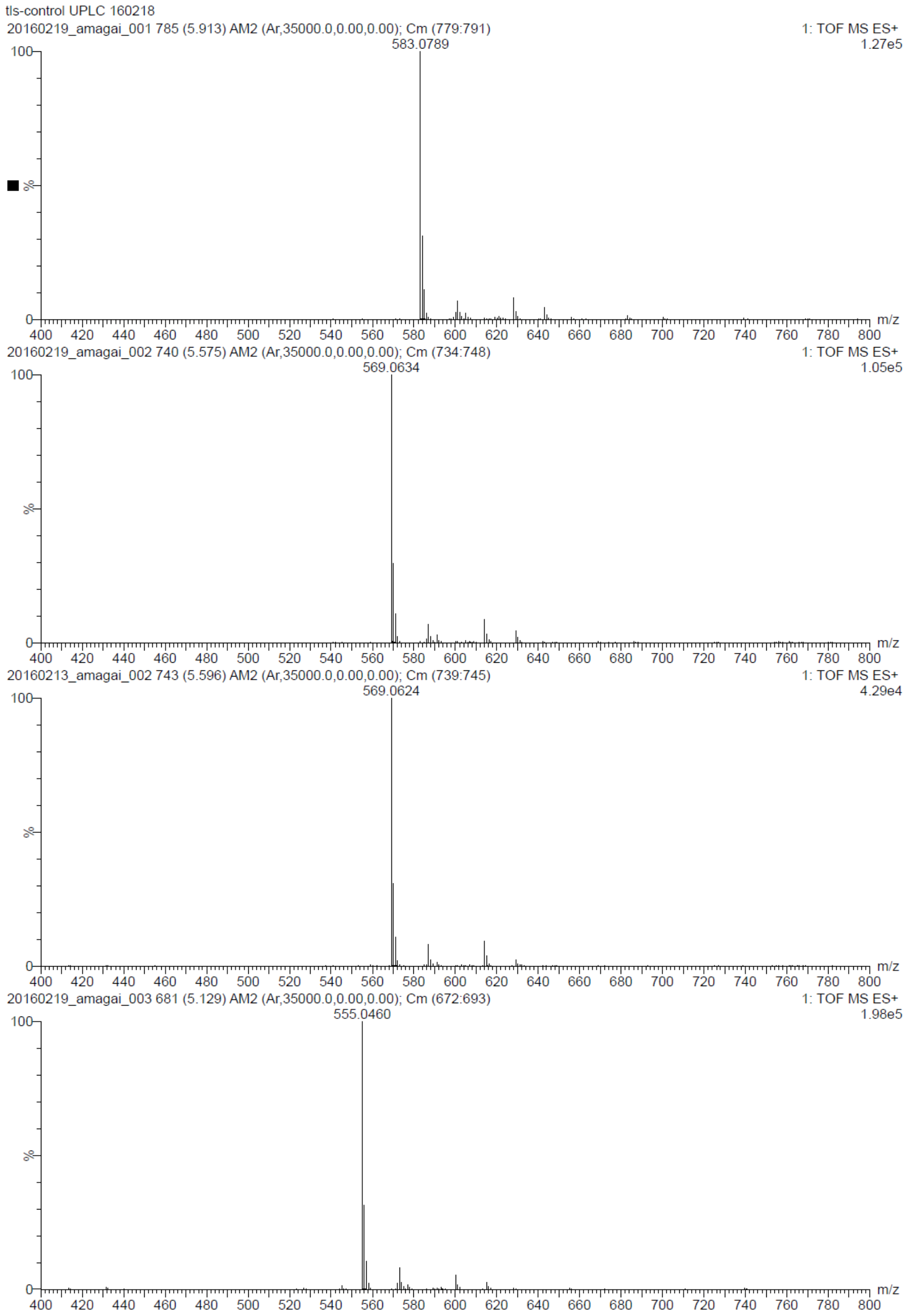

Figure S8. HPLC/ESI mass spectra of the **1**, **2**, **3**, and **4** samples at each peak position. Top to bottom: Retention time = 5.91 min, 5.59 min, 5.59 min, and 5.13 min, respectively.

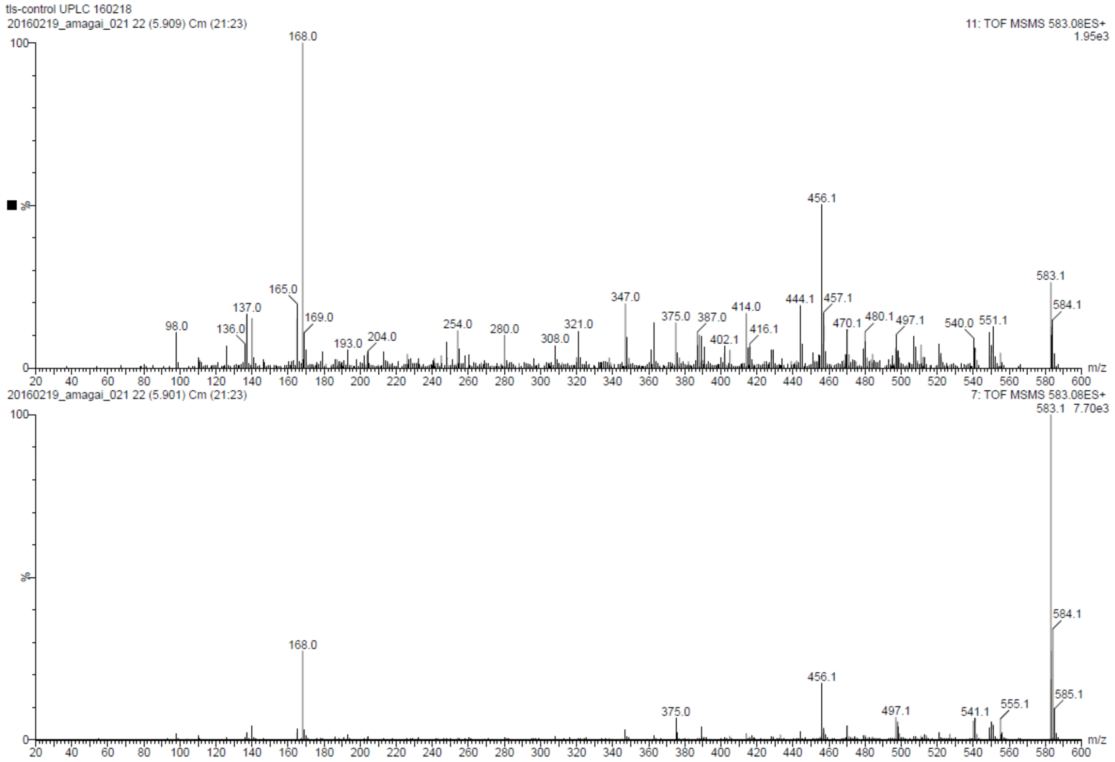

Figure S9. CID MS/MS spectra of the protonated molecule of **1** at a nominal collision-energy setting of 42 V (top) or 34 V (bottom).

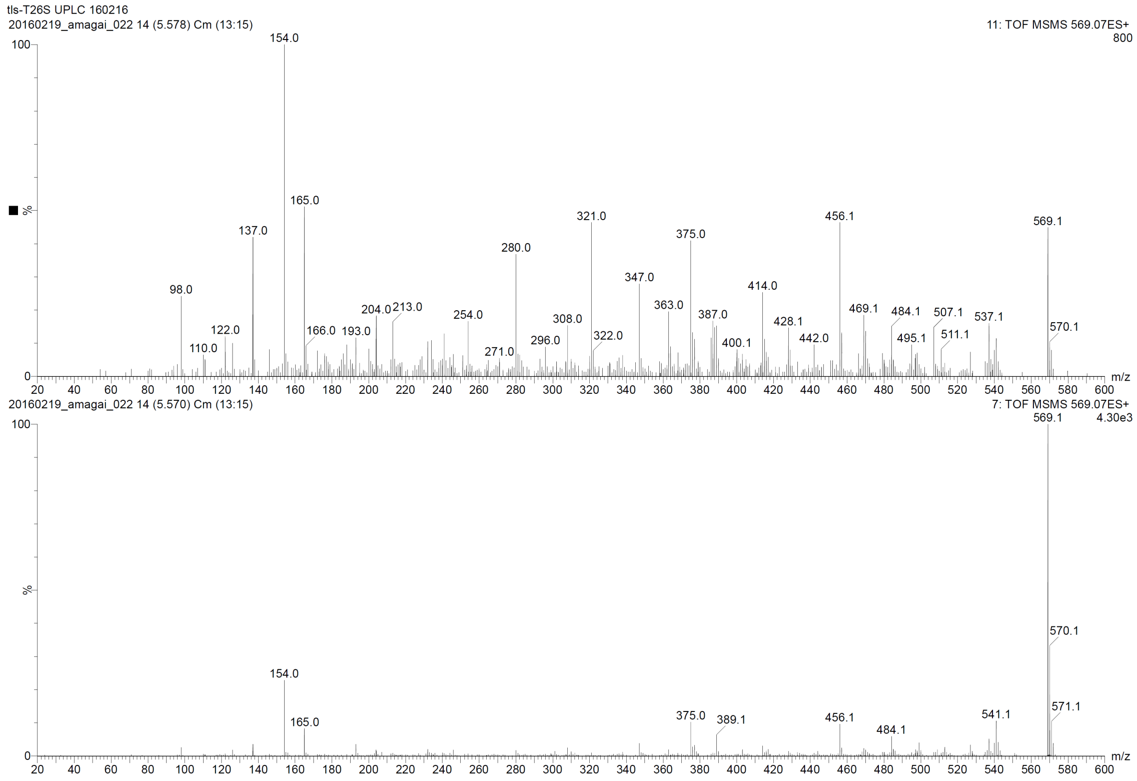

Figure S10. CID MS/MS spectra of the protonated molecule of **2** at a nominal collision-energy setting of 42 V (top) or 34 V (bottom).

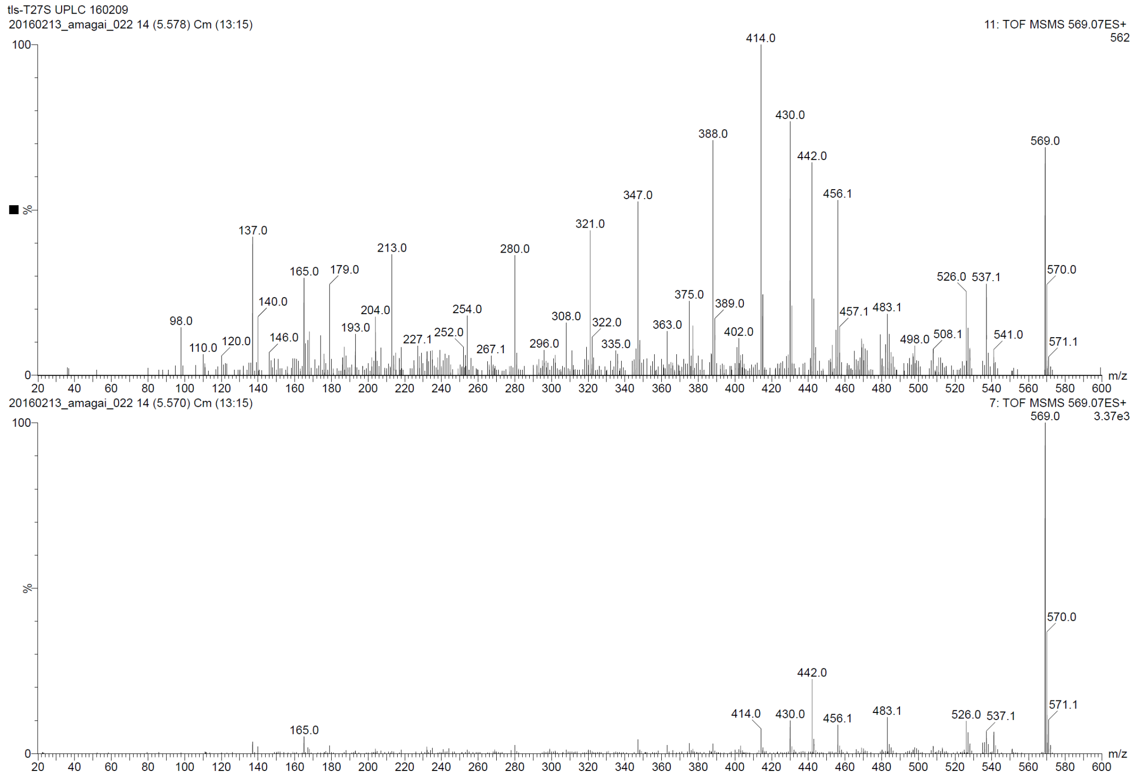

Figure S11. CID MS/MS spectra of the protonated molecule of **3** at a nominal collision-energy setting of 42 V (top) or 34 V (bottom).

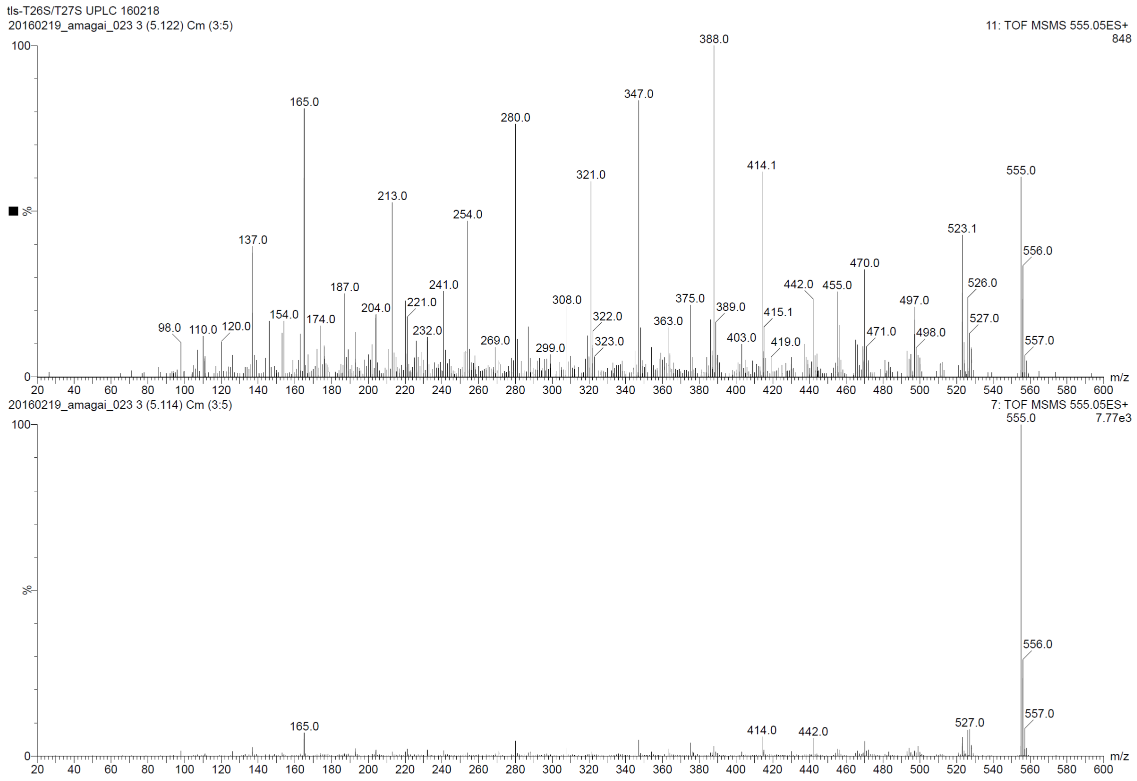

Figure S12. CID MS/MS spectra of the protonated molecule of **4** at a nominal collision-energy setting of 42 V (top) or 34 V (bottom).

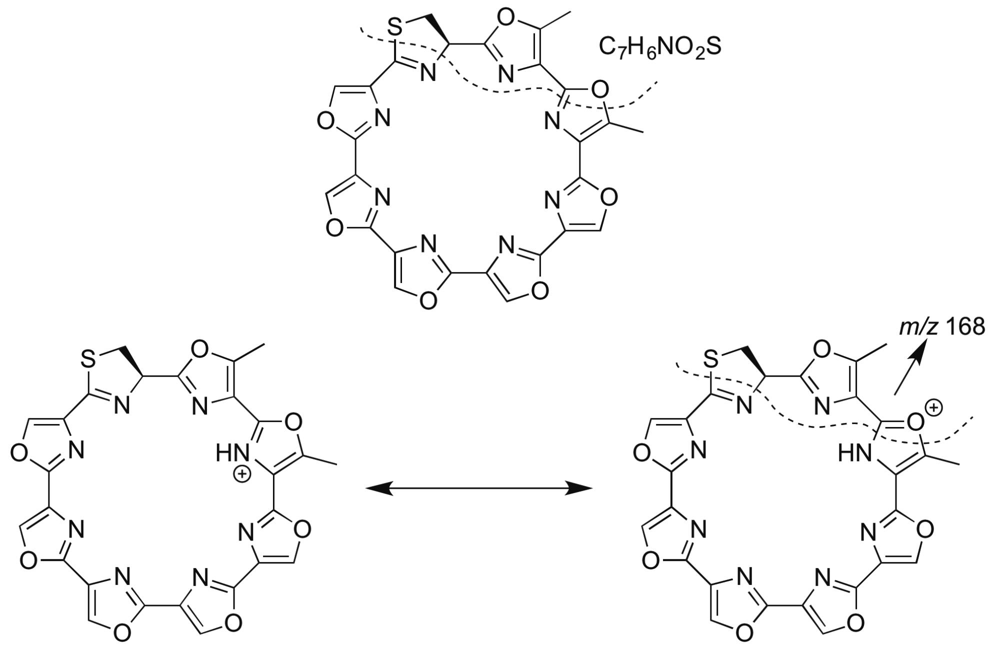

Figure S13. Proposed structural assignment of the characteristic product ion at *m/z* 168.

Table S1. Putative functions of ORFs in the 1 biosynthetic gene cluster

Gene designation	Size [bases]	Encoded protein homologue (GenBank accession number), % Positives/% Identity	Putative function of deduced polypeptide
tlsA	2,274	hypothetical protein (WP_051433755), 99/99	MMPL family transporter
tlsB	660	hypothetical protein M877_37625 (EST18691), 99/99	transcriptional regulator
tlsC	129	hypothetical protein M877_37630 (EST18692), 100/100	precursor peptide (CTTSSSSSS)
tlsD	924	hypothetical protein (WP_023543552), 98/98	SAM-dependent methyltransferase
tlsE	798	hypothetical protein (WP_023543553), 99/99	MaoC dehydratase
tlsF	984	hypothetical protein (WP_023543554), 98/96	acyltransferase
tlsG	780	SDR family oxidoreductase (WP_023543555), 98/97	3-oxoacyl-ACP reductase
tlsH	261	hypothetical protein (WP_023543556), 100/100	acyl carrier protein
tlsI	1,233	hypothetical protein (WP_023543557), 100/100	3-oxoacyl-ACP synthase
tlsJ	777	SDR family oxidoreductase (WP_023543558), 100/100	3-oxoacyl-ACP reductase
tlsK	1,011	hypothetical protein (WP_023543559), 98/98	epimerase
tlsL	1,287	hypothetical protein (WP_023543560), 99/97	PLP-demendent aminotransferase
tlsM	577	hypothetical protein (WP_023543561), 93/91	polyketide cyclase
tlsN	1,191	hypothetical protein M877_37685 (EST18703), 98/98	acyl-CoA dehydrogenase
tlsO	918	hypothetical protein (WP_023543563), 97/96	hypothetical protein
tlsP	1,803	hypothetical protein (WP_023543564), 96/95	FMN-dependent nitroreductase
tlsQ	2,184	hypothetical protein (WP_023543565), 97/96	YcaO protein
tlsR	783	hypothetical protein (WP_023543566), 98/97	hypothetical protein
tlsS	1,182	hypothetical protein M877_37710 (EST18708), 98/97	peptidase M16
tlsT	1,071	hypothetical protein (WP_023543568), 98/97	peptidase M16C
tlsU	876	hypothetical protein (WP_023543569), 98/98	MOSC domain containing protein

Table S2. Vectors and strains used in this study

Vectors/Plasmids	Characteristic(s)	Source/Reference
pKU492Acos_*aac(3)IV*	Vector for cloning of the putative telomestatin biosynthetic gene cluster	[19]
pKU460::*rpsJp-aac(3)I*	Plasmid for preparing other promoter cassette constructs	[18]
pKU460::*sav2794p-aac(3)I*	Plasmid for promoter cassette preparation	this study
pKU460::*sav2794p-FRT-aac(3)IV-FRT*	Plasmid for promoter cassette preparation	this study
pKU460::*ssmp*p-aac(3)I*	Plasmid for promoter cassette preparation	this study
pKU460::*ssmp*p-FRT-aac(3)IV-FRT*	Plasmid for promoter cassette preparation	this study
pKU460::*olmRp-aac(3)I*	Plasmid for promoter cassette preparation	this study
pKD78	Vector for λ-RED recombinase expression	CGSC
pGEM-3zf	Vector for amplifying beta-lactamase gene	Applied Biosystems
pKD13_*aac(3)IV*	Vector for preparing *FRT-aac(3)IV-FRT* gene	this study
pKU503Dtls_P4-K8	BAC clone including the *tls* cluster	this study
pKU503DoliP15-N14	BAC clone including the oligomycin biosynthetic gene cluster	this study
pKU492Acos::*xylAp*::*tls*_cluster	Plasmid for heterologous expression of the *tls* cluster in *S. avermitilis* SUKA	this study
pKU492Acos::*olmRp*::*tls*_cluster	Plasmid for heterologous expression of the *tls* cluster in *S. avermitilis* SUKA	this study
pKU492Acos::*sav2794p*::*tls*_cluster	Plasmid for heterologous expression of the *tls* cluster in *S. avermitilis* SUKA	this study
pKU592A_*aph(3')II* (pKU592A)	Vector for cloning of the putative telomestatin biosynthetic gene cluster	this study
pKU592A::*sav2794p-tls*	Plasmid for heterologous expression of the *tls* cluster in *S. avermitilis* SUKA	this study
pKU592A::*sav2794p*::*tls*_cluster Δ*tlsA*	Plasmid for gene inactivation experiments	this study
pKU592A::*sav2794p*::*tls*_cluster Δ*tlsB*	Plasmid for gene inactivation experiments	this study
pKU592A::*sav2794p*::*tls*_cluster Δ*tlsAB*	Plasmid for gene inactivation experiments	this study
pKU592A::*sav2794p*::*tls*_cluster Δ*tlsD-N*	Plasmid for gene inactivation experiments	this study
pKU592A::*sav2794p*::*tls*_cluster Δ*tlsD-N* Δ*tlsSTU*	Plasmid for gene inactivation experiments	this study
pKU592A::*sav2794p*::*tls*_cluster Δ*tlsO*	Plasmid for gene inactivation experiments	this study
pKU592A::*sav2794p*::*tls*_cluster Δ*tlsP*	Plasmid for gene inactivation experiments	this study
pKU592A::*sav2794p*::*tls*_cluster Δ*tlsQ*	Plasmid for gene inactivation experiments	this study
pKU592A::*sav2794p*::*tls*_cluster Δ*tlsR*	Plasmid for gene inactivation experiments	this study
pKU592A::*sav2794p*::*tls*_cluster Δ*tlsS*	Plasmid for gene inactivation experiments	this study
pKU592A::*sav2794p*::*tls*_cluster Δ*tlsT*	Plasmid for gene inactivation experiments	this study
pKU592A::*sav2794p*::*tls*_cluster Δ*tlsST*	Plasmid for gene inactivation experiments	this study
pKU592A::*sav2794p*::*tls*_cluster Δ*tlsU*	Plasmid for gene inactivation experiments	this study
pKU592A_*aac(3)IV*	Vector for cloning of the putative telomestatin biosynthetic gene cluster	this study
pKU592A_*aac(3)IV*::*sav2794p*::*tls*_cluster	Plasmid for heterologous expression of the *tls* cluster in *S. avermitilis* SUKA	this study
pKU592A_*aac(3)IV*::*sav2794p*::*tls*_cluster T26S	Plasmid for heterologous expression of the *tls* derivative gene cluster in *S. avermitilis* SUKA	this study
pKU592A_*aac(3)IV*::*sav2794p*::*tls*_cluster T27S	Plasmid for heterologous expression of the *tls* derivative gene cluster in *S. avermitilis* SUKA	this study
pKU592A_*aac(3)IV*::*sav2794p*::*tls*_cluster T26S/T27S	Plasmid for heterologous expression of the *tls* derivative gene cluster in *S. avermitilis* SUKA	this study
pKU592A_*aac(3)IV*::*sav2794p*::*tls_cluster* Δ*tlsCcore*	Plasmid for gene inactivation experiments	this study

Table S2 (continued)

Strain		
E. coli		
NEB10β	Host for constructing the BAC library	New England Biolabs
DH5α	Host for general cloning	Takara
stellar	Host for general cloning	Takara
BW25113	Host for λ-RED recombination	CGSC
GM2929 *hsdS::Tn10*	Methylation-deficient donor for preparing plasmids introduced into *S. avermitilis* SUKA	[18]
Streptomyces		
Streptomyces anulatus 3533-SV4	Telomestatin-producing strain	[1]
S. avermitilis SUKA17	Host for heterologous expression	[18]
S. avermitilis SUKA22	Host for heterologous expression	[19]
SUKA17 (pKU492Acos::*xylAp*::*tls*_cluster)	SUKA17 integrated with the *xylAp*::*tls*_cluster	this study
SUKA17 (pKU492Acos::*olmRp*::*tls*_cluster)	SUKA17 integrated with the *olmRp*::*tls*_cluster	this study
SUKA17 (pKU492Acos::*sav2794*::*tls*_cluster)	SUKA17 integrated with the *sav2794p*::*tls*_cluster	this study
SUKA17 (pKU592A::*sav2794p*::*tls*_cluster)	SUKA17 integrated with the *sav2794p*::*tls*_cluster	this study
SUKA22 (pKU592A::*sav2794p*::*tls*_cluster Δ*tlsCcore*)	SUKA17 integrated with the *sav2794p*::*tls*_cluster with a TlsC core peptide deletion	this study
SUKA22 (pKU592A::*sav2794p*::*tls*_cluster Δ*tlsA*)	SUKA22 integrated with the *sav2794p*::*tls*_cluster with a *tlsA* gene deletion	this study
SUKA22 (pKU592A::*sav2794p*::*tls*_cluster Δ*tlsB*)	SUKA22 integrated with the *sav2794p*::*tls*_cluster with a *tlsB* gene deletion	this study
SUKA22 (pKU592A::*sav2794p*::*tls*_cluster Δ*tlsAB*)	SUKA22 integrated with the *sav2794p*::*tls*_cluster with deletion of the *tlsA* and *tlsB* genes	this study
SUKA22 (pKU592A::*sav2794p*::*tls*_cluster Δ*tlsD-N*)	SUKA22 integrated with the *sav2794p*::*tls*_cluster with a deletion between the *tlsD* and *tlsN* genes	this study
SUKA22 (pKU592A::*sav2794p*::*tls*_cluster Δ*tlsD-N* Δ*tlsSTU*)	SUKA22 integrated with the *sav2794p*::*tls*_cluster with a deletion of between the *tlsD* and *tlsN* genes, *tlsS, tlsT,* and *tlsU* genes	this study
SUKA22 (pKU592A::*sav2794p*::*tls*_cluster Δ*tlsO*)	SUKA22 integrated with the *sav2794p*::*tls*_cluster with a *tlsO* gene deletion	this study
SUKA22 (pKU592A::*sav2794p*::*tls*_cluster Δ*tlsP*)	SUKA22 integrated with the *sav2794p*::*tls*_cluster with a *tlsP* gene deletion	this study
SUKA22 (pKU592A::*sav2794p*::*tls*_cluster Δ*tlsQ*)	SUKA22 integrated with the *sav2794p*::*tls*_cluster with a *tlsQ* gene deletion	this study
SUKA22 (pKU592A::*sav2794p*::*tls*_cluster Δ*tlsR*)	SUKA22 integrated with the *sav2794p*::*tls*_cluster with a *tlsR* gene deletion	this study
SUKA22 (pKU592A::*sav2794p*::*tls*_cluster Δ*tlsS*)	SUKA22 integrated with the *sav2794p*::*tls*_cluster with a *tlsS* gene deletion	this study
SUKA22 (pKU592A::*sav2794p*::*tls*_cluster Δ*tlsT*)	SUKA22 integrated with the *sav2794p*::*tls*_cluster with a *tlsT* gene deletion	this study
SUKA22 (pKU592A::*sav2794p*::*tls*_cluster Δ*tlsST*)	SUKA22 integrated with the *sav2794p*::*tls*_cluster with a deletion of *tlsS* and *tlsT* genes	this study
SUKA22 (pKU592A::*sav2794p*::*tls*_cluster Δ*tlsU*)	SUKA22 integrated with the *sav2794p*::*tls*_cluster with a *tlsU* gene deletion	this study
SUKA17 (pKU592A_*aac(3)IV*::*sav2794p*::*tls*_cluster)	SUKA17 integrated with the *sav2794p-tls* cluster	this study
SUKA17 (pKU592A_*aac(3)IV*::*sav2794p*::*tls*_cluster TlsC T26S)	SUKA17 integrated with the *sav2794p*::*tls*_cluster encoding the TlsC T26S mutation	this study
SUKA17 (pKU592A_*aac(3)IV*::*sav2794p*::*tls*_cluster TlsC T27S)	SUKA17 integrated with the *sav2794p*::*tls*_cluster encoding the TlsC T27S mutation	this study

Table S3. Primers used in this study. Bold characters show *Spe*I sites; underlined characters show homologous sequence arms for λ-RED recombination or In-Fusion cloning.

Primers	Sequences (5'-3')	
492_F	GGATCCTCTAGAGTCGACCTG	For construction of pKU492Acos_*aac(3)IV*::*xylAp*::*tls*_cluster
492xylA_R	ATGGCGGCTCCTTGCTTGCT	For construction of pKU492Acos_*aac(3)IV*::*xylAp*::*tls*_cluster
tls-3frg_F	GAACTGATCGGCGAA**ACTAGT**GGCCCAACCTCCTTCTGGA	For construction of pKU492Acos_*aac(3)IV*::*xylAp*::*tls*_cluster
tls-3frg_R	CGACTCTAGAGGATCCATCTACAACCTCGCGTACAC	For construction of pKU492Acos_*aac(3)IV*::*xylAp*::*tls*_cluster
tls-5frg_F	GCAAGGAGCCGCCATATGATCATCGCCGGACCAGGT	For construction of pKU492Acos_*aac(3)IV*::*xylAp*::*tls*_cluster
tls-5frg_R	GAAGGAGGTTGGGCC**ACTAGT**TTCGCCGATCAGTTCCGGGA	For construction of pKU492Acos_*aac(3)IV*::*xylAp*::*tls*_cluster
olmRp-aacI_F	GCTTATGTCCAATTCCAGTTCGTCCGTCGCTTCG	For construction of pKU460::*olmRp-aac(3)I*
olmRp-460_R	CTAGAGGATCCTCATATGCCGTACGAGATCCTCCA	For construction of pKU460::*olmRp-aac(3)I*
460-olmRp-F	GATCTCGTACGGCATATGAGGATCCTCTAGAGTCG	For construction of pKU460::*olmRp-aac(3)I*
aacI-olmRp_R	GCGACGGACGAACTGGAATTGGACATAAGCCTGTTC	For construction of pKU460::*olmRp-aac(3)I*
olmRp-tls_R	TCTTGAACGCTCAACGACTCCCCCAGCTGACCTGGTCCGGCG<br>ATGATCATATGGAATCGCGAAACACCCCGCA	For construction of pKU492Acos_*aac(3)IV*::*olmRp*::*tls*_cluster by λ-RED recombination
sav2794p-tls_R	CTCTTGAACGCTCAACGACTCCCCCAGCTGACCTGGTCCGGC<br>GATGATCATATGTGACTCCTTCTGCGTGG	For construction of pKU492Acos_*aac(3)IV*::*sav2794p*::*tls*_cluster by λ-RED recombination
Univ2_F	TCCTTTAGCAGCCCTTGCGCCCT	For construction of pKU492Acos_*aac(3)IV*::*promoter*::*tls*_cluster by λ-RED recombination
pKD13-Apr_F	CAGAGCGCTTTTGAAGCTCACGCTGCCGCAAGCACTCAGGAT<br>CGCTATAATGACCCCGAA	For substitution of *aph(3')II* in pKD13 with *aac(3)IV*
pKD13-Apr_R	ATAGGAACTTCGGAATAGGAACTTCAAGATCCCCTTATTATC<br>ATGAGCTCAGCCAATCGA	For substitution of *aph(3')II* in pKD13 with *aac(3)IV*
460aacI-aacIV_F	AAGCGCGTTACGCCGTGGGTCGATGTTTGATGTTATGGAGCA<br>GCAACGATTACCGTTCGTATAGCATACA	For antibiotic-marker substitution of pKU460::*promoter-aac(3)I* vector with *FRT-aac(3)IV-FRT*
460aacI-aacIV_R	AGTGCTTGCGGCAGCGTGAAGCTAGCGATCTCGGCTTGAACG<br>AATTGTTATACCGTTCGTATAATGTAT	For antibiotic-marker substitution of pKU460::*promoter-aac(3)I* vector with *FRT-aac(3)IV-FRT*
del-tlsA_F	TCCTTTAGCAGCCCTTGCGCCCTGAGTGCTTGCGGCAGCGTG<br>AAGCTAGCTGTAGGCTGGAGCTGCTTC	For construction of pKU592A::*sav2794p*::*tls*_cluster Δ*tlsA* and Δ*tlsAB* by λ-RED recombination
del-tlsA_R	GTGTCACCGTCTTCGTGCGTGCTCGTACATTCGGTGGGAAAG<br>GTGTGCATATGTGACTCCTTCTGCGTGG	For construction of pKU592A*sav2794p*::*tls*_cluster Δ*tlsA* by λ-RED recombination
del-tlsB_F	CGGCACTACGTAATCGACCACATGCGAGTCAAATCGGGGGCC<br>CTGCTGTGTGTAGGCTGGAGCTGCTTC	For construction of pKU592A::*sav2794p*::*tls*_cluster Δ*tlsB* by λ-RED recombination
del-tlsB_R	TCCAGGCTGGAGATCTGCTCGTCGACAATGTCGAACGTGATG<br>TCTGCCATATGTGACTCCTTCTGCGTGG	For construction of pKU592A::*sav2794p*::*tls*_cluster Δ*tlsB* and Δ*tlsAB* by λ-RED recombination
del-tlsO_F	GGCGGCCCGGCGCTAGATTCGAAGACGTGAATACTTCTGAGC<br>CAGCGGAATGTAGGCTGGAGCTGCTTC	For construction of pKU592A::*sav2794p*::*tls*_cluster Δ*tlsO* by λ-RED recombination
del-tlsO_R	CGGGCCGCGAAGCCCTGCTCCGTCACGCAGCGCAGCATGTCC<br>TCGTCCACATGTGACTCCTTCTGCGTGG	For construction of pKU592A::*sav2794p*::*tls*_cluster Δ*tlsO* by λ-RED recombination
del-tlsP_F	TGTGCGCACCGCACGCCCCCGCACGCCAGGAATGGGCGGAGG<br>TCTGAGCGTGTAGGCTGGAGCTGCTTC	For construction of pKU592A::*sav2794p*::*tls*_cluster Δ*tlsP* by λ-RED recombination
del-tlsP_R	TCGTCGTCCAGTACGTGAACGCTCACACGTACGCGCTGTGTC<br>TCCGTCATATGTGACTCCTTCTGCGTGG	For construction of pKU592A::*sav2794p*::*tls*_cluster Δ*tlsP* by λ-RED recombination
del-tlsQ_F	TCTATCTGACACTGCTCGGCCCGCGACGCACAGGGGATGAGG<br>CCGCATGATGTAGGCTGGAGCTGCTTC	For construction of pKU592A::*sav2794p*::*tls*_cluster Δ*tlsQ* by λ-RED recombination
del-tlsQ_R	TCGTTGCGGACGACGGACAGGGAGGGGTACGCGAGGAATGAG<br>GACGCCATATGTGACTCCTTCTGCGTGG	For construction of pKU592A::*sav2794p*::*tls*_cluster Δ*tlsQ* by λ-RED recombination
del-tlsR_F	CCGACAACCCGTATCCGCACCCGTTGCCCTGAACGTGGCAAG<br>GAGCTCCGTGTAGGCTGGAGCTGCTTC	For construction of pKU592A::*sav2794p*::*tls*_cluster Δ*tlsR* by λ-RED recombination
del-tlsR_R	AGGTGGCGGGGCCCGGTCGGGTCGCGGGGCCCGGTGGTCAGG<br>ACGGGCACATGTGACTCCTTCTGCGTGG	For construction of pKU592A::*sav2794p*::*tls*_cluster Δ*tlsR* by λ-RED recombination
del-tlsS_F	AGACCGTGGTGACCCTGGTCTCCACCGAGGCCGGCCGGTGCC<br>CGTCCTGATGTAGGCTGGAGCTGCTTC	For construction of pKU592A::*sav2794p*::*tls*_cluster Δ*tlsS* and Δ*tlsST* by λ-RED recombination
del-tlsS_R	TGTTCGCCGGGAACGCTGTTGGCGAGGACCGGTGCGCCGGGG<br>GCCGTCATATGTGACTCCTTCTGCGTGG	For construction of pKU592A::*sav2794p*::*tls*_cluster Δ*tlsS* by λ-RED recombination
del-tlsT_F	GGTGGCGGCAGGGGTTCGCCGCCGCCCTGCCCGAAGGCCCGG<br>CGACATGATGTAGGCTGGAGCTGCTTC	For construction of pKU592A::*sav2794p*::*tls*_cluster Δ*tlsT* by λ-RED recombination
del-tlsT_R	ATGCTCTTGACGGGGTAGCGCCAGATCTCTCGTATCGCGAAC<br>CTGTTCACATGTGACTCCTTCTGCGTGG	For construction of pKU592A::*sav2794p*::*tls*_cluster Δ*tlsT* and Δ*tlsST* by λ-RED recombination
del-tlsU_F	ACGTCGCACGGCGCCATATCGGCTCGGACCAACCGGGAGGGG<br>ACTTGTGATGTAGGCTGGAGCTGCTTC	For construction of pKU592A::*sav2794p*::*tls*_cluster Δ*tlsU* by λ-RED recombination
del-tlsU_R	AGGCGCGGGGCACGGCGGGCGGCGGCATCGGTCGCGTGACGC<br>CGCCCTGTATTCCGGGGATCCGTCGACC	For construction of pKU592A::*sav2794p*::*tls*_cluster Δ*tlsU* by λ-RED recombination

Table S3 (continued)

delFAS-bla_F	CGCGGCGCCACTGTCCGCGTCACCGCGTGAGGCCGGGACGGAGCGAACAC**ACTAGT**TTTCTACGGGGTCTGACGC	For substitution of *tlsD-tlsN* genes with the *bla* gene by λ-RED recombination
delFAS-bla_R	ATCTAGCGCCGGGCCGCCCGTGATCCGATACGTCTTTCCGTAGTACGGAA**ACTAGT**TTCAAATATGTATCCGCTCATGAGAC	For substitution of *tlsD-tlsN* genes with the *bla* gene by λ-RED recombination
NoScar-delFAS_F	CGCGTCACCGCGTGAGGCCGGGACGGAGCGAACACTTCCGTACTACGGAAAGACGTATCGGATCACGGGC	For construciton of pKU592A::*sav2794p*::*tls*_cluster Δ*tlsD-N* by Gibson assembly
NoScar-delFAS_R	GCCCGTGATCCGATACGTCTTTCCGTAGTACGGAAGTGTTCGCTCCGTCCCGGCCTCACGCGGTGACGCG	For construciton of pKU592A::*sav2794p*::*tls*_cluster Δ*tlsD-N* by Gibson assembly
bla-tlsCcore_F	GATTTTCCGCGCCGGAATTACTCGAAGTCGACGTCGGAGATGTCGAGAGA**ACTAGT**TTCAAATATGTATCCGCTCATGAGAC	For construction of a *bla* gene cassette for deletion of *tlsC* core peptide coding sequence
bla-tlsCcore_R	TTGTCGACGAGCAGATCTCCAGCCTGGAGTCGATCGGCGTCGAGGAAGGC**ACTAGT**TTTCTACGGGGTCTGACGC	For construction of a *bla* gene cassette to delete the *tlsC* core peptide coding sequence
T26S_F	AGCCTGGAGTCGATCGGCGTCGAGGAAGGCTGCTCGACCTCTTCGTCGAGCAGCTCTCTCGACATCTCCGACGTCGACTTCGAG	For construction of a telomestatin derivative-producing gene cluster by Gibson assembly
T26S_R	CTCGAAGTCGACGTCGGAGATGTCGAGAGAGCTGCTCGACGAAGAGGTCGAGCAGCCTTCCTCGACGCCGATCGACTCCAGGCT	For construction of a telomestatin derivative-producing gene cluster by Gibson assembly
T27S_F	AGCCTGGAGTCGATCGGCGTCGAGGAAGGCTGCACGAGCTCTTCGTCGAGCAGCTCTCTCGACATCTCCGACGTCGACTTCGAG	For construction of a telomestatin derivative-producing gene cluster by Gibson assembly
T27S_R	CTCGAAGTCGACGTCGGAGATGTCGAGAGAGCTGCTCGACGAAGAGCTCGTGCAGCCTTCCTCGACGCCGATCGACTCCAGGCT	For construction of a telomestatin derivative-producing gene cluster by Gibson assembly
T26S/T27S_F	AGCCTGGAGTCGATCGGCGTCGAGGAAGGCTGCAGCAGCTCTTCGTCGAGCAGCTCTCTCGACATCTCCGACGTCGACTTCGAG	For construction of a telomestatin derivative-producing gene cluster by Gibson assembly
T26S/T27S_R	CTCGAAGTCGACGTCGGAGATGTCGAGAGAGCTGCTCGACGAAGAGCTGCTGCAGCCTTCCTCGACGCCGATCGACTCCAGGCT	For construction of a telomestatin derivative-producing gene cluster by Gibson assembly

Table S4. Predicted and measured accurate masses for the protonated molecules of each compound.

compound	molecular formula	calculated *m/z* for $[M+H]^+$	measured *m/z*	error (ppm)
**1**	$C_{26}H_{14}N_8O_7S$	583.0784	583.0789	0.9
**2**	$C_{25}H_{12}N_8O_7S$	569.0628	569.0634	1.1
**3**	$C_{25}H_{12}N_8O_7S$	569.0628	569.0624	-0.7
**4**	$C_{24}H_{10}N_8O_7S$	555.0471	555.0460	-2.0

Table S5. Measured accurate product-ion masses in the CID spectrum of 1 (nominal collision-energy setting of 42 V) and their assignments to possible chemical formulae.

measured mass	relative abundance	formula	calculated mass	error (ppm)
168.0118	100	$C_7H_6NO_2S$	168.0119	-0.6
347.0533	25.09	$C_{16}H_7N_6O_4$	347.0529	1.2
375.048	21.86	$C_{17}H_7N_6O_5$	375.0478	0.5
414.0588	21.49	$C_{19}H_8N_7O_5$	414.0587	0.2
444.0694	23.94	$C_{20}H_{10}N_7O_6$	444.0693	0.2
456.0694	64.83	$C_{21}H_{10}N_7O_6$	456.0693	0.2
583.0781	35.26	$C_{26}H_{15}N_8O_7S$	583.0784	-0.5

Materials and Methods

DNA manipulation

PCR conditions were as follows. In reactions using Phusion DNA polymerase, initial denaturation at 98 °C for 30 s was followed by 30 cycles of amplification (98 °C, 10 s; 55–72 °C, 30 s; and 72 °C, 30 s/kb) and a final incubation at 72 °C for 5 min. When using the Expand High Fidelity PLUS PCR system, initial denaturation at 94 °C for 2 min was followed by 10 cycles of amplification (98 °C, 30 s; 55–68 °C, 30 s; and 72 °C, 1 min/kb) and 20 additional cycles of amplification (98 °C, 30 s; 55–68 °C, 30 s; and 72 °C, 1 min/kb, with 10 s added to the elongation time for each successive cycle), with a final incubation at 72 °C for 7 min. When using Pfu Ultra II Fusion HS DNA polymerase, initial denaturation at 95 °C for 20 s was followed by 30 cycles of amplification (95 °C, 20 s; 55–72 °C, 30 s; and 72 °C, 15 s/kb) and a final incubation at 72 °C for 3 min. DNA amplification was optimized by varying the amplification time and annealing temperature (Phusion DNA polymerase or Pfu turbo Ultra II: from 55–72 °C; Expand High Fidelity PLUS PCR system: from 55–68 °C). Emerald Amp premix or Ex *Taq* (Takara Bio, Inc.) was used for colony PCR. PCR primers were designed using Primer3 (http://frodo.wi.mit.edu) on Geneious (version 8.1.7, created by Biomatters, available at http://www.geneious.com/). The oligo nucleotide primers were purchased from Hokkaido System Sciences Co., Ltd. (Tokyo Japan) or Eurofins Genomics (Tokyo Japan).

Preparation of 1 and its derivatives, and analysis of their distribution

S. avermitilis SUKA (pKU592Acos::*sav2794p-tls*) and transformants with introduced point mutations in the *tlsC* gene (encoding the T26S, T27S, and T26S/T27S peptides), were cultured in 10 mL of TSB medium containing 5 μg/mL of apramycin at 28 °C, with rotation at 240 rpm, for 3 d. The pre-culture (0.5 mL) was then transferred to 70 mL of 0.3× BPS medium (15 flasks each, total 1.05 L) and cultured at 28 °C with rotation at 150 rpm for 4 d. The resultant cells were collected by centrifugation, washed with 50 mM HEPES-NaOH (pH 7.0), sonicated on ice (TOMY UD-200, 30 s operation, 10 times, 3 min/interval), and extracted 3 times with 15 mL of *n*-BuOH. The organic layer was evaporated to dryness. Each *n*-BuOH extract (500 mg) was separated by

silica-gel column chromatography (60Å 70–230 mesh [Merck], 25 g, 2 × 20 cm) after equilibration with $CHCl_3$/MeOH (2:1). The materials were developed in 60 mL of MeOH followed by 180 mL of $CHCl_3$/MeOH (2:1). The resultant partially purified **1** and its derivatives were further purified using an ACQUITY UPLC (Waters), equipped with an ACQUITY UPLC BEH C_{18} column (1.7 μm, 2.1 × 50 mm; Waters). Derivatives of **1** were eluted as follows: flow rate of 0.2 mL/min; [0.1% TFA in water] as solvent A; [3:2 mixture of 0.1 % TFA in CH_3CN and 2-PrOH] as solvent B; 10–90% B linear gradient in 10 min. Compound **1** and its derivatives (**2**–**4**) were collected based on absorbance at 260 nm.

To examine the distribution of **1** in *S. avermitilis* SUKA22 and Δ*tlsA* transformants, cells and medium were separated by centrifugation at 5,000 × *g* for 30 min, and **1** was extracted as described above.

High-resolution HPLC/MS and HPLC/MS/MS analysis

HPLC-purified samples of **1**, a 6-desmethylated derivative (**2**) of **1**, a 9-desmethylated derivative (**3**) of **1**, and a 6,9-didesmethylated derivative (**4**) of **1** were subjected to high-resolution HPLC/MS and HPLC/MS/MS analysis to confirm their structures. Electrospray ionization (ESI)-time-of-flight (TOF) MS and MS/MS spectra were obtained with a Synapt G2 mass spectrometer (Waters, Manchester, UK) equipped with an ACQUITY UPLC system (Waters). The HPLC conditions were as follows: ACQUITY UPLC BEH C_{18} column (2.1 mm × 50 mm); flow rate of 0.2 mL/min; [0.1% TFA in water] as solvent A; [3:2 mixture of 0.1 % TFA in CH_3CN and 2-PrOH] as solvent B; 10% B for 1 min, and then 10–95% B over 8.5 min. The mass spectrometer was operated in high-resolution mode (full width at half maximum resolution of 40,000) for mass analysis, or in resolution mode (full width at half maximum resolution of 20,000) for MS/MS analysis. The ESI source was operated in positive-ion mode with a 3.0 kV sprayer and a 60 V cone voltage. To measure the masses accurately, leucine enkephalin was introduced into the ion source simultaneously, using an auxiliary sprayer to generate internal-lock mass signals at *m*/*z* 556.2711 and 278.1141 (protonated molecules and b_3 ions) when operating in MS mode, or *m*/*z* 425.1825 and 120.0813 (b_4 and F ions) when operating in MS/MS mode. For collision-induced

dissociation (CID) MS/MS experiments, a DC offset (corresponding to the nominal collision-energy setting) of 8–60 V was applied between the mass-resolving quadrupole and the succeeding collision cell, where argon was introduced for collisional activation.

Chemical derivatization and Gas chromatography (GC) /MS analysis

Aliquot crude samples containing FA297 were dissolved in 1:1 mixture of MeOH and 2-PrOH and dried down in micro glass tubes by using a vacuum centrifuge. The samples in each glass tubes were placed in autosampler vials, derivatized with *N*-methyl-*N*-(trimethylsilyl)trifluoroacetamide (MSTFA) or *N*-methylbistrifluoroacetamide (MBTFA), both purchased from Wako Pure Chemical Industries (Osaka, Japan), prior to GC/MS analysis. Authentic alkyl amines including *n*-butylamine, 2-aminooctane, and *N*-methyloctadecylamine, all purchased from Tokyo Chemical Industry (Tokyo, Japan) were derivatized in the same manner to obtain reference standards. A JEOL (Akishima, Japan) JMS-T100GCV time-of-flight mass spectrometer equipped with an electron-ionization (EI) / field-ionization (FI) combination source and an Agilent (Santa Clara, California) 7890A gas chromatograph was operated for GC/MS analysis under the following conditions. Carrier gas (He) flow rate was 1.5 ml/min; ZB-1MS column (Phenomenex, Torrance, California) with 0.25 μm film thickness 0.32 mm i.d. x 30 m; 250 °C splitless injection port; column oven temperature 70 °C for initial 4 min and increased in 30 °C/min to 325 °C and kept for 1.5 min. Outlet of the column was placed in the combination ion source kept at 200 °C for EI mode or 60 °C for FI mode. Mass spectra (full width at half maximum resolution of 8,000) were recorded in every 0.2 sec (70 eV EI mode) or in every 0.4 sec (FI mode). Post-run drift compensation on *m*/*z* axis was carried out by using column bleed siloxane peak at *m*/*z* 207.0325.

Structural characterization of FA297

When a crude sample of **1** was subjected to high-resolution HPLC/MS analysis, FA297 was eluted around 9.6 min (Fig. S4, top panel). At least, two components that show protonated molecules at *m*/*z* 298 were clearly visible. The molecular formula of both main and minor components was determined to be $C_{20}H_{43}N$ based on accurate mass

measurements and isotopic peak pattern (Fig. S4, lower panels). Crude samples of **2**, **3**, and **4** showed the same peaks corresponding to FA297 (data not shown).

The molecular formula of FA297 does not allow presence of any unsaturation and possible candidate structures were limited to alkyl amines (linear or branched, but non-cyclic). In order to characterize FA297 further, a crude sample containing FA297 was subjected to chemical derivatization and GC/MS analysis. As shown in the top and 2nd panel of Fig. S5, two peaks corresponding to the molecular ions of TFA or TMS derivatives of FA297 were observed in FI mode. As no peaks corresponding to molecular ions of underivatized FA297 were observed (data not shown), tertiary amine structures were ruled out. The EI mass spectra of both TMS derivatives (bottom panels of Fig. S5) show fragment ions characteristic to TMS derivatives (*m/z* 354 and 73) and the base peak at *m/z* 116, as well. Accurate mass of the peak (found 116.0897; calcd. for $C_5H_{14}NSi$ 116.0896) showed the fragment ion was attributable to an iminium ion ($[C_2H_5N\text{-}TMS]^+$) generated by α-cleavage of TMS-derivatized amines. However, no further structural information on alkyl moiety could be extracted from the spectra.

Confirmation of the molecular formulae and structures of 1, 2, 3, and 4

Figure S5 shows UV and extracted-ion chromatograms for **1–4**. Each sample showed a distinct peak at an *m/z* ratio corresponding to the predicted protonated molecules. The mass spectrum for each peak is shown in Fig. S8. The measured masses were matched to the calculated *m/z* values for protonated molecules for each compound, within an error range of 2 ppm (Table S5). Figure S9 shows CID MS/MS spectra for the protonated molecules of **1**, and Table S5 shows the measured masses and assigned formulae for the major product ions. In general, interpretation of the product-ion spectra of macrocycles is a formidable task because i) at least two bonds must be cleaved to generate fragment ions, and ii) the initial ring-cleavage site(s) is often unknown or cannot be identified easily. In addition, this particular type of compound has repeated structural units, which is a trait that further complicates spectral interpretation. However, a characteristic sulphur-containing product ion was observed at *m/z* 168. The most abundant peak in the higher collision-energy spectrum, *m/z* 168, also represented one of the major product ions in the lower-energy spectrum. Moreover, the *m/z* 168 peak was

clearly visible even in the spectrum recorded at a nominal collision-energy setting of 22 V, in which roughly 98% the of precursor ions were unfragmented (data not shown). Therefore, it is plausible that this product ion was closely related to the primary-fragmentation channels from the precursor ions. Thus, it is also possible that this product ion well reflects the structure of the precursor protonated molecules, or of the compound itself. Although elucidation of detailed fragmentation mechanisms and/or ion structures was beyond the scope of the current study, tentative assignment of this *m/z* 168 product ion was attempted. Based on the measured accurate masses (Table S5), the elemental composition of the *m/z* 168 product was deduced to be $C_7H_6NO_2S$. As mentioned above, this ion was plausibly generated without extensive skeletal rearrangement. Given the known structure of **1**, the $C_7H_6NO_2S$ product ion is most likely attributable to the upper region of the neutral structure drawn in Fig. S13. A possible precursor ion structure that could have generated this product ion is proposed in Fig. S13.

The CID MS/MS spectra for the protonated molecules of **2** (Fig. S10) clearly showed that *m/z* 168 in **1** was shifted to *m/z* 154. The measured accurate mass of this characteristic product ion was 153.9966 (error 1.9 ppm; calculated mass for $C_6H_4NO_2S$ = 153.9963) and supported the predicted structure of **2** (T26S derivative of **1**), in which the methyl group on the oxazole ring adjacent to the thiazoline was missing. However, virtually no *m/z* 168 or *m/z* 154 peaks were observed in the CID spectra of **3** (T27S derivative of **1**) or **4** (T26S/T27S derivative of **1**, as shown in Figs. S11 and S12). These rather big changes in spectral patterns are quite consistent to the proposed structures of these metabolites. The methyl groups on the second oxazole ring were adjacent to the cleavage site that would generate *m/z* 168 from **1** (see bottom right structure of Fig. S13) or *m/z* 154 from **2**. As the methyl groups on second oxazole were missing in **3** and **4**, the possible initial cleavage site became not favorable any longer and other competing fragmentation channels dominated.
